# Strand specific RNA-sequencing and membrane lipid profiling reveals growth phase-dependent cold stress response mechanisms in *Listeria monocytogenes*

**DOI:** 10.1371/journal.pone.0180123

**Published:** 2017-06-29

**Authors:** Patricia Hingston, Jessica Chen, Kevin Allen, Lisbeth Truelstrup Hansen, Siyun Wang

**Affiliations:** 1 Department of Food, Nutrition, and Health, The University of British Columbia, Vancouver, British Columbia, Canada; 2 National Food Institute, Technical University of Denmark, Kongens Lyngby, Denmark; National Renewable Energy Laboratory, UNITED STATES

## Abstract

The human pathogen *Listeria monocytogenes* continues to pose a challenge in the food industry, where it is known to contaminate ready-to-eat foods and grow during refrigerated storage. Increased knowledge of the cold-stress response of this pathogen will enhance the ability to control it in the food-supply-chain. This study utilized strand-specific RNA sequencing and whole cell fatty acid (FA) profiling to characterize the bacterium’s cold stress response. RNA and FAs were extracted from a cold-tolerant strain at five time points between early lag phase and late stationary-phase, both at 4°C and 20°C. Overall, more genes (1.3×) were suppressed than induced at 4°C. Late stationary-phase cells exhibited the greatest number (n = 1,431) and magnitude (>1,000-fold) of differentially expressed genes (>2-fold, p<0.05) in response to cold. A core set of 22 genes was upregulated at all growth phases, including nine genes required for branched-chain fatty acid (BCFA) synthesis, the osmolyte transporter genes *opuCBCD*, and the internalin A and D genes. Genes suppressed at 4°C were largely associated with cobalamin (B12) biosynthesis or the production/export of cell wall components. Antisense transcription accounted for up to 1.6% of total mapped reads with higher levels (2.5×) observed at 4°C than 20°C. The greatest number of upregulated antisense transcripts at 4°C occurred in early lag phase, however, at both temperatures, antisense expression levels were highest in late stationary-phase cells. Cold-induced FA membrane changes included a 15% increase in the proportion of BCFAs and a 15% transient increase in unsaturated FAs between lag and exponential phase. These increases probably reduced the membrane phase transition temperature until optimal levels of BCFAs could be produced. Collectively, this research provides new information regarding cold-induced membrane composition changes in *L*. *monocytogenes*, the growth-phase dependency of its cold-stress regulon, and the active roles of antisense transcripts in regulating its cold stress response.

## Introduction

The human pathogen *Listeria monocytogenes* continues to pose a challenge in the food industry, where it is known to contaminate ready-to-eat foods and to grow during refrigerated storage. Ingestion of *L*. *monocytogenes* by susceptible individuals can cause potentially fatal food-borne infections with mortality rates as high as 40% reported [1]. The ubiquitous nature of this pathogen makes it difficult to eliminate from our food systems however, post-processing levels of *L*. *monocytogenes* contamination are often low [2–4] and unlikely to cause disease [5, 6]. Furthermore, the risks associated with *L*. *monocytogenes* contamination can be addressed by heating foods to an appropriate temperature [7]. Therefore, the ability of *L*. *monocytogenes* to grow to unsafe levels in ready-to-eat foods during the shelf-life of products, pose the greatest concern to consumer health.

When subjected to low temperatures, all living cells will experience similar challenges that stem from a reduction in molecular dynamics, which leads to decreased rates of diffusion and structural changes to molecular structures [8]. Cold-stress adaptation mechanisms among microbes include membrane compositional changes, compatible solute uptake, and the synthesis of nucleic acid stabilization proteins, DNA-unwinding enzymes, and general stress response and cold shock proteins [9, 10]. Currently, most of our knowledge regarding bacterial cold stress response (CSR) mechanisms comes from the model organisms *Escherichia coli* and *Bacillus subtilis*. However, unlike *L*. *monocytogenes*, neither of these bacteria can multiply at temperatures close to 0°C. In the last decade, *L*. *monocytogenes* outbreaks associated with ready-to-eat foods in both North America and Europe have increased interest in the specific mechanisms employed by this psychrotrophic pathogen to adapt and grow at low temperatures.

The CSR of *L*. *monocytogenes* has been studied using microarrays [11–13], quantitative real-time PCR (qPCR) [14–17], mutagenesis [18–24] and other genetic and proteomic techniques [25–27]. Collectively, these methods have identified a large pool of genes with putative or known roles in cold tolerance. However, although a small portion of qPCR-based studies have looked at the differential expression (DE) of select genes during the early stages of the *L*. *monocytogenes* CSR (i.e., lag phase) [16, 17], global transcriptome studies have only been conducted on cold-adapted exponential- and stationary-phase cells to date, leaving much to be discovered regarding the initial CSR of *L*. *monocytogenes*. Furthermore, since CSRs become more pronounced in response to more dramatic changes in temperature, many studies have focused on abrupt temperature down shifts from 37°C to 15°C or lower [11, 14, 15, 17, 28]. Such conditions may not represent the food industry, where more commonly *L*. *monocytogenes* is typically transferred from an ambient-temperature environment to a food product or plant environment that is maintained at 4–10°C. Though exposure to ambient temperature (20–25°C) can also be considered a low temperature stress [15, 29], the maximum growth rate (μ_max_) of *L*. *monocytogenes*’ is approximately 10× faster at 20°C than at 4°C whereas there is much less of a difference between 15°C and 37°C (~4.5×) [30]. Moreover, at 37°C, the transcriptional landscape of *L*. *monocytogenes* undergoes a drastic change in preparation for intracellular survival [31].

Recent advances in molecular and sequencing technologies now allow us to detect several forms of non-coding RNA (ncRNA). Studies of ncRNA have increased our understanding of gene regulation and opened a new area in bacterial stress response research.

ncRNAs exist in several different forms and thus participate in a wide range of functions. Most ncRNAs can be divided into three main categories: 1) Cis-regulatory RNAs, 2) trans-encoded small RNAs (sRNAs), and 3) antisense RNAs (asRNAs) [32]. Cis-regulatory RNAs are located at the 5’-ends of mRNA and fold into alternative structures in response to physicochemical cues. These transcripts are often referred to as riboswitches or thermosensors. One of the best-known examples in *L*. *monocytogenes* is the riboswitch that blocks the translation of the major virulence regulator *prfA* at low temperatures (<30°C) [33]. Trans-encoded sRNAs, on the other hand, are not located adjacent to their target and share only limited complementarity allowing them to regulate multiple mRNAs. Lastly, asRNAs are transcribed from the DNA strand opposite of a gene and thus have perfect complementarity. asRNAs can be short (<100 nt) or long (>1000 nt) and in some cases correspond to the 5’- or 3’- extension of an mRNA transcribed from an adjacent gene [34]. The prevalence of genes with reported asRNA ranges from 20–75% depending on the organism [35, 36]. To date, more than 100 asRNAs have been described in *L*. *monocytogenes* [31, 36–38]. Compared with the numbers reported for other bacteria this number seems rather low. However, it will presumably rise with further study.

The objective of this study was to gain a comprehensive view of the *L*. *monocytogenes* CSR, with an emphasis on elucidating mRNA and asRNA expression patterns across multiple growth phases following cold stress. To gain a better understanding of the mechanisms employed by *L*. *monocytogenes* to grow during refrigerated storage, we aimed to characterize the CSR from cells during the initial lag phase following cold stress, throughout exponential growth and long-term stability at low temperatures, using conditions similar to those present in a food-contamination scenario. To accomplish these goals, strand-specific RNA sequencing and membrane lipid profiling were conducted on *L*. *monocytogenes* cell cultures at five distinct growth phases, following a downshift in temperature from 20°C to 4°C. As the phenotypic and genetic properties of *L*. *monocytogenes* isolates differ, we chose to elucidate the CSR mechanisms of a strain with relevance to the food industry and consumer health: a fast cold-growing, serotype 1/2a, food plant isolate containing full length versions of important virulence genes: *inlA*, *inlB*, *inlC*, *prfA*, *plcA*, *hly*, *mpl*, *actA*, *plcB*. Here, we report novel findings regarding cold-induced membrane compositional changes in *L*. *monocytogenes*, the growth phase-dependent cold-stress regulon, and the active roles of antisense transcripts in regulating the CSR of this pathogen.

## Materials and methods

### Culture conditions and time point selection

**Fig 1:**
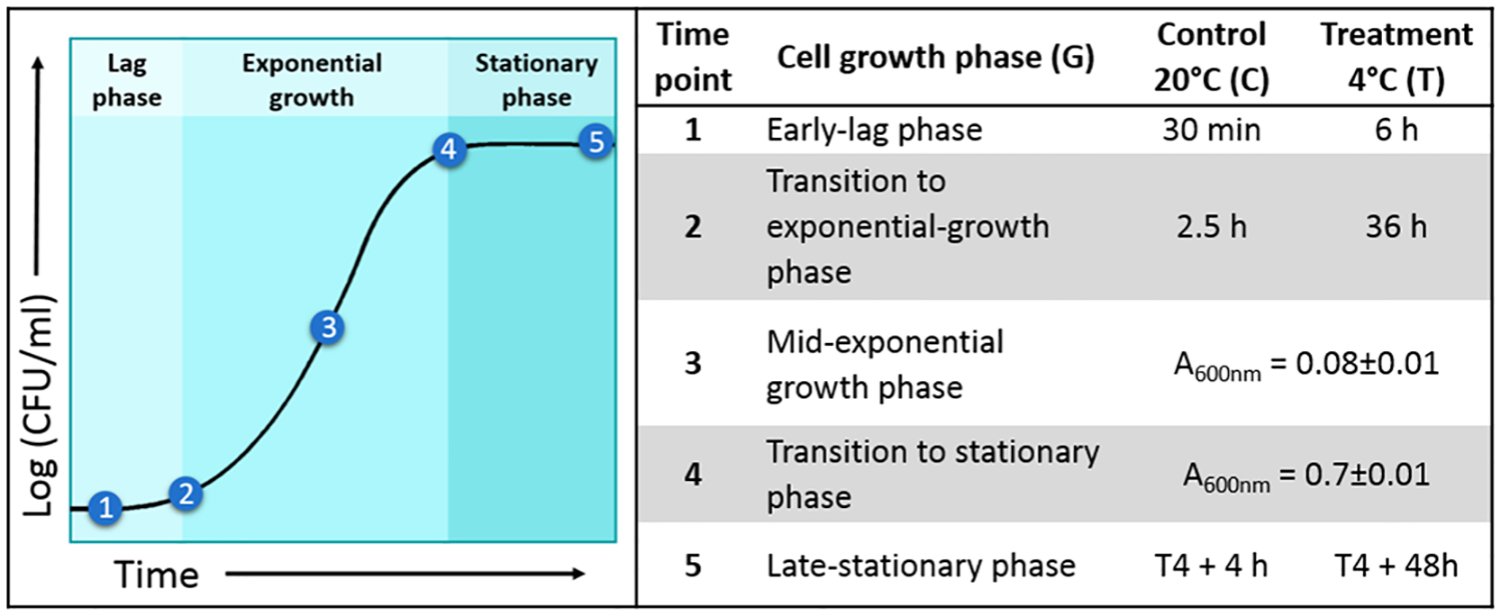
Cell growth phase sampling strategy at 4°C and 20°C.

A previously evaluated *L*. *monocytogenes* environmental isolate (BioSample SAMN05256775) from a food-processing plant in British Columbia (Lm1, serotype 1/2a, sequence type 7) was selected for use in this study [39]. The strain displayed enhanced cold tolerance (μ_max_ at 4°C) relative to a large collection of isolates [39]. Bacterial cultures were grown for 24 h in brain heart infusion broth (BHIB; Difco, Fisher Scientific, Canada) at 20°C, re-suspended in pre-tempered BHIB at a cell density of 10^7^ CFU/ml and incubated at 4 or 20°C. RNA and lipids were extracted at five time points from cells grown at 4°C (treatment, T) and 20°C (control, C). Three biological replicates were conducted for each treatment and control sample. Each time point corresponded to a specific growth phase (G) (Fig 1). The time points were as follows: G1 –early lag phase, G2 –transition to exponential growth phase, G3 –mid-exponential growth phase, G4 –transition to stationary phase, and G5 –late-stationary phase. G1 corresponded to 20% of the lag phase duration at each temperature. The timing was determined by modelling previously obtained growth curve data using the Baranyi and Roberts model [40] in DMfit (v3.5) (http://browser.combase.cc/DMFit.aspx) [30]. G2 was marked by a doubling in cell numbers, confirmed using plate counts. G3 corresponded to mid-exponential growth (10^8^ CFU/ml), and G4 corresponded to the transition to stationary phase; both time points were identified spectrophotometrically (A_600nm_) and confirmed with plate counts. Lastly, G5 was selected to correspond to G4 plus 8× the lag phase duration (h) at each temperature.

### Fatty acid analysis

At each of the five growth phases for cells grown at both 20°C and 4°C, cultures were pelleted (10–45 mg net weight), rinsed twice with one ml phosphate buffered saline (PBS, Fisher Scientific), and stored at -80°C. One sample per time point was collected. The frozen pellets were later sent to MIDI labs (Microbial ID, Inc., Newark DE, USA) where the cell lipids were extracted, followed by methylation of the fatty acids (FAs), and then loaded onto a gas chromatograph for analysis. Fatty acid methyl ester (FAME) profiles were then generated and analyzed using Sherlock^®^ pattern recognition software.

### RNA isolation and sequencing

At each sample point, growth in cultures was stopped by adding 10% phenol:chloroform (Fisher Scientific) in ethanol solution pre-chilled to -80°C in a 1:10 volume to the sample. The tubes were vortexed briefly and then centrifuged immediately for 10 min at 4,696×*g* and 0°C. The supernatants were removed and the resulting pellets were stored at -80°C for less than 2 weeks. Total RNA was isolated and purified using the PowerMicrobiome^™^ RNA Isolation kit (MO BIO Laboratories, CA, USA) per the manufacturer’s protocol. RNA integrity numbers (RINs) were determined using the 2100 Bioanalyzer (Agilent, CA, USA). Samples with a RIN between 9.7 and 10 and an RNA concentration >100 ng/μl were sent to Genome Québec (Montréal, QC, Canada) for rRNA-depleted Illumina TruSeq RNA library prep and TruSeq stranded total RNA 100 bp paired-end sequencing on the Illumina HiSeq 2000 platform.

### RNA-seq data analysis

Sequencing quality was assessed using FastQC [41], and Illumina adapter sequences and low-quality base pairs were removed using default parameters in Trimmomatic v 0.36 [42]. After the removal of low-quality reads, 22–39 million reads remained for each dataset. Reads were mapped to the complete sequenced genome of *L*. *monocytogenes* EGD (NCBI RefSeq NC_022568.1) using Bowtie 2 v 2.2.6 [43] and allowing zero mismatches. *L*. *monocytogenes* EGD was selected as the reference genome as it allowed for the highest percent of successfully mapped reads compared to the more commonly employed reference strains EGDe and 10403S. The mapping efficiency ranged from 97.8–99.6% for individual reads and 92.1–95.6% for successfully paired reads. BAM alignment files were used as input for read counting using featureCounts v 1.5.0-p1 [44]. The default counting mode ‘union’ was used, and separate count files were generated for sense and antisense transcripts. Differential expression (DE) analyses were performed using DESeq2 [45] in R v 3.1.1 [46], and the DE was reported as log_2_ fold changes. *p*-values were adjusted by the DESeq2 default Benjamini-Hochberg (BH) adjustment method and genes with a >2-fold (>1 log_2_) change in expression and an adjusted *p*-value < 0.05 were considered as DE. A principle component analysis (PCA) was conducted using DESeq2 to determine the overall reproducibility of our RNA-seq biological replicates. To evaluate the overall transcription levels for individual genes at both 4°C and 20°C, raw read counts were normalized based on library size. No p-values were calculated for genes with low mean normalized counts, zero counts, or extreme outlier counts.

### Clustering of gene expression profiles

Clustering of genes with similar expression patterns across the five growth phases, was performed using the Mfuzz package [47] in R. Default filtering and standardization methods (based on standard deviation) were applied to the log_2_ data from all five growth phases. Soft clustering was then performed using the fuzzy *c*-means algorithm and the following parameters: c = 20, m = 1.25. Genes were assigned to clusters of given expression patterns across the growth-phases based on having a membership value >0.5.

### Functional categorization of differentially expressed genes

To investigate the roles of DE genes at each growth phase, we determined the overrepresentation of molecular and biological pathways (Fisher exact test, p<0.05) using SmartTables based on the BioCyc database (https://biocyc.org/) [48]. SmartTables were also used to map *L*. *monocytogenes* EGD genes to the equivalent genes in *L*. *monocytogenes* 10403S for which the BioCyc database contains transcription regulation and gene ontology information. Additional enrichment analyses were then performed (Fisher’s exact test, p<0.05) for gene ontology (GO) terms, and genes regulated by certain transcription regulators (i.e., σ^B^, PrfA, RpoD, VirR, MogR, CodY, HrcA and CtsR).

### Quantitative PCR validation of RNA-seq data

RNA-seq results were validated using qPCR amplification of three genes: 1) *leuA* which exhibited >4-fold higher expression at 4°C at all five growth phases, 2) *cspB* which exhibited >4-fold lower expression at 4°C at all growth phases, and 3) *recJ* which was chosen as a reference gene because it showed very little variation in expression across all growth phases at either temperature (S2 Table). Up to 1 μg of RNA from each of our 30 samples was reverse transcribed using the QuantiTect Reverse Transcription Kit (Qiagen, Valencia, CA) per the manufacturer’s protocol. Primers were designed using Primer3 Plus (Table 1) and our draft whole genome sequence for Lm1 (GenBank Accession number GCA_001709805.1). qPCR was conducted in a CFX96 Touch^™^ Real-Time PCR Detection System (BioRad) using SsoAdvanced ^™^ Universal SYBR Green ^®^ Supermix (BioRad). The relative expression levels of *leuA* and *cspB* were calculated using the 2^-ΔΔCT^ method [49], with *recJ* as the reference gene.

**Table 1:** Primers used for quantitative PCR validation of RNA-seq data.

Gene abbr.	Primer sequence	Opt. Anneal. Temp	Source
*cspB*	Fw-CAAACAGGTACAGTTAAATGGTTTA	55°C	[17]
	Rv-ACGATTTCAAATTCAACGCTTTGA		
*leuA*	Fw-TTGTCGGGTATGCCTGTTCC	55°C	This study
	Rv-GGGTTTTTCAGCACGCCATC		
*recJ*	Fw-CTCGACCGGCAATTGTGTTG	55°C	This study
	Rv- GTCCACACTTCGACCAGACC		

### Accession numbers

FastQ files of HiSeq runs were deposited into the NCBI Sequence Read Archive under BioProject PRJNA384077.

## Results and discussion

### mRNA transcriptome of *L*. *monocytogenes* cold-stress response

A total of 11–17 million paired-end mRNA reads per sample were successfully assigned to *L*. *monocytogenes* EGD open reading frames (ORFs). The number of reads mapped to each EGD ORF ranged from 0 to 630,000. No counts were observed for 27 ORFs, of which, 26 were confirmed to be absent in our strain, leaving one gene (*LMON*_0476) with no detectable expression at 20°C or 4°C. Overall, >99% of EGD ORFs were expressed by Lm1 at 20 and 4°C. This percentage is in line with the findings of Toledo-Arana et al. [31], who reported that *Listeria* spp. express more than 98% of their ORFs at 30°C and 37°C.

**Fig 2:**
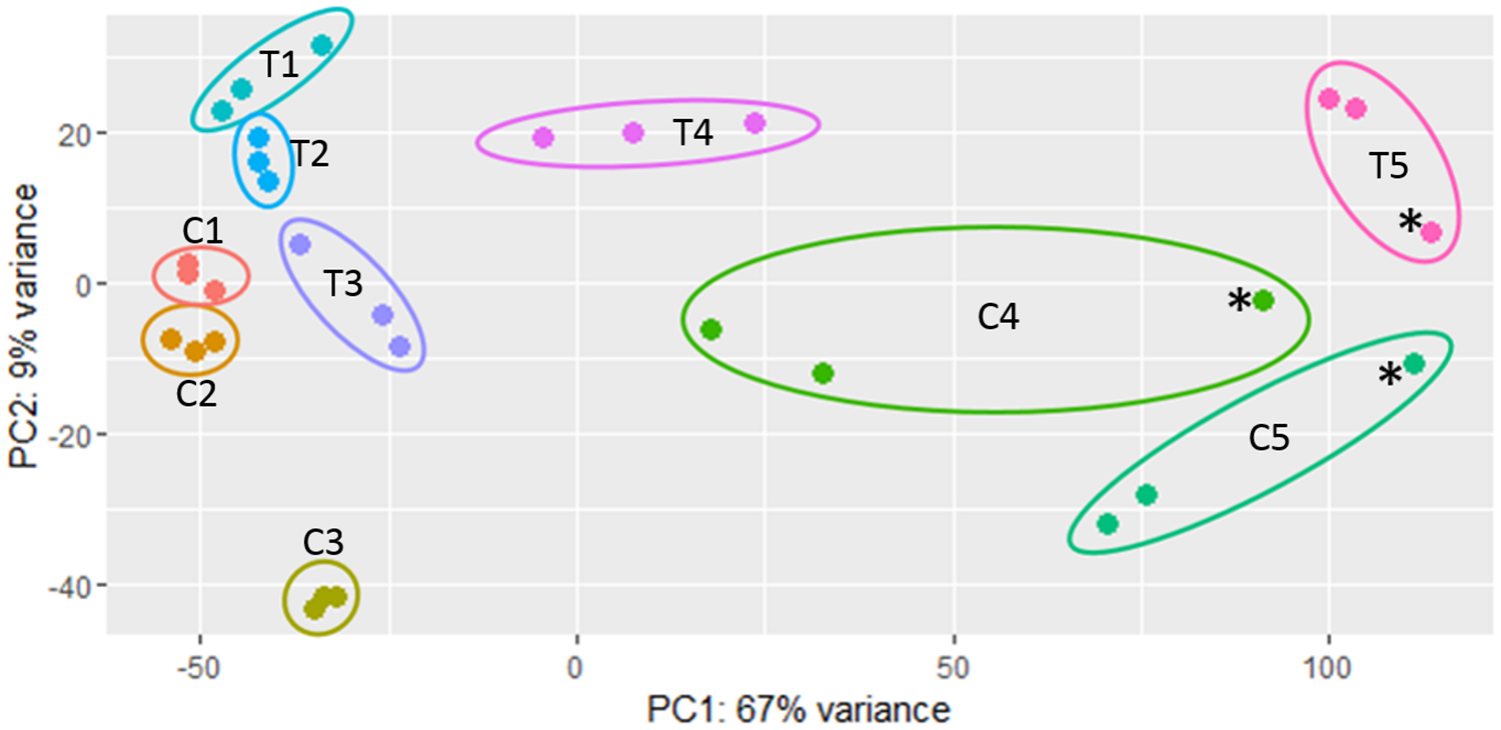
Principle component analysis plot of RNA sequencing biological replicates. C1 through C5 refer to the *L*. *monocytogenes* control cultures grown at 20°C, and T1 through T5 represent the treated cultures grown at 4°C (see Fig 1 for information about the sampling points). Samples marked with * were excluded from our analyses due to an abundance of outlier data points.

Three of the 30 sequenced samples were excluded from further analysis as they were deemed to be outliers as visualized in the PCA plot in Fig 2. The excluded samples included one biological replicate each from the T4, C4, and C5 treatments. Two of the three samples were from G4 which represents the transition from exponential growth to stationary phase. Given the short window of time available for extracting RNA from this very specific physiological phase, it is likely that these samples were processed either too early or too late relative to the other two biological replicates. The excluded C5 sample belonged to the same biological replicate as the excluded C4 sample. The PCA plot (Fig 2) also shows that the level of transcriptome variance is much greater (67%) among growth phases than between the two temperatures (9%), highlighting the importance of growth phase selection in determining the outcomes of an experiment.

**Fig 3:**
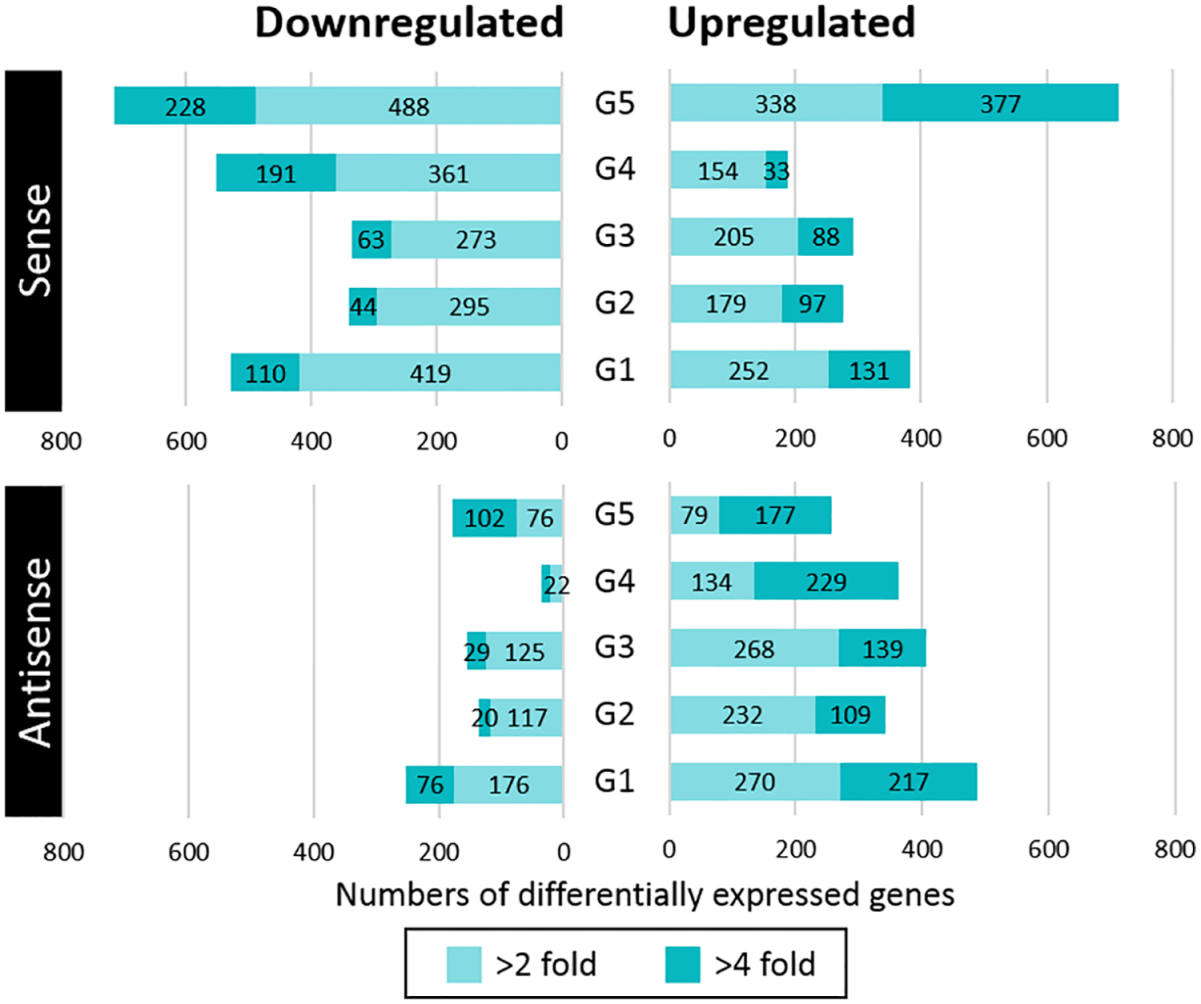
Numbers of *L*. *monocytogenes* sense and antisense RNAs differentially expressed at five growth phases in response to cold stress (4°C relative to 20°C). Differential expression (DE) analyses were performed using DESeq2 and DE was reported as log_2_ fold changes. Genes with a >2-fold change (> 1 log_2_) in expression and an adjusted *p*-value < 0.05 were considered DE. See Fig 1 for information about growth phases.

Overall, 1.3× more genes were suppressed than induced in *L*. *monocytogenes* under cold stress (Fig 3). This finding contrasts with the results reported in a microarray study conducted by Chan et al. [11], where 30–40% fewer genes were downregulated than upregulated in *L*. *monocytogenes* at 4°C compared to 37°C. However, in agreement with their work, we observed that cold-adapted stationary-phase cells exhibited the greatest number of DE genes (G5, Fig 3). From here on, the DE of genes at 4°C vs. 20°C will be discussed with respect to the growth phases (G1–G5) and C1–C5 and T1–T5 will be reserved for when referring to overall levels of transcription or changes in the membrane lipid profiles that occur at 20°C and 4°C, respectively.

**Fig 4:**
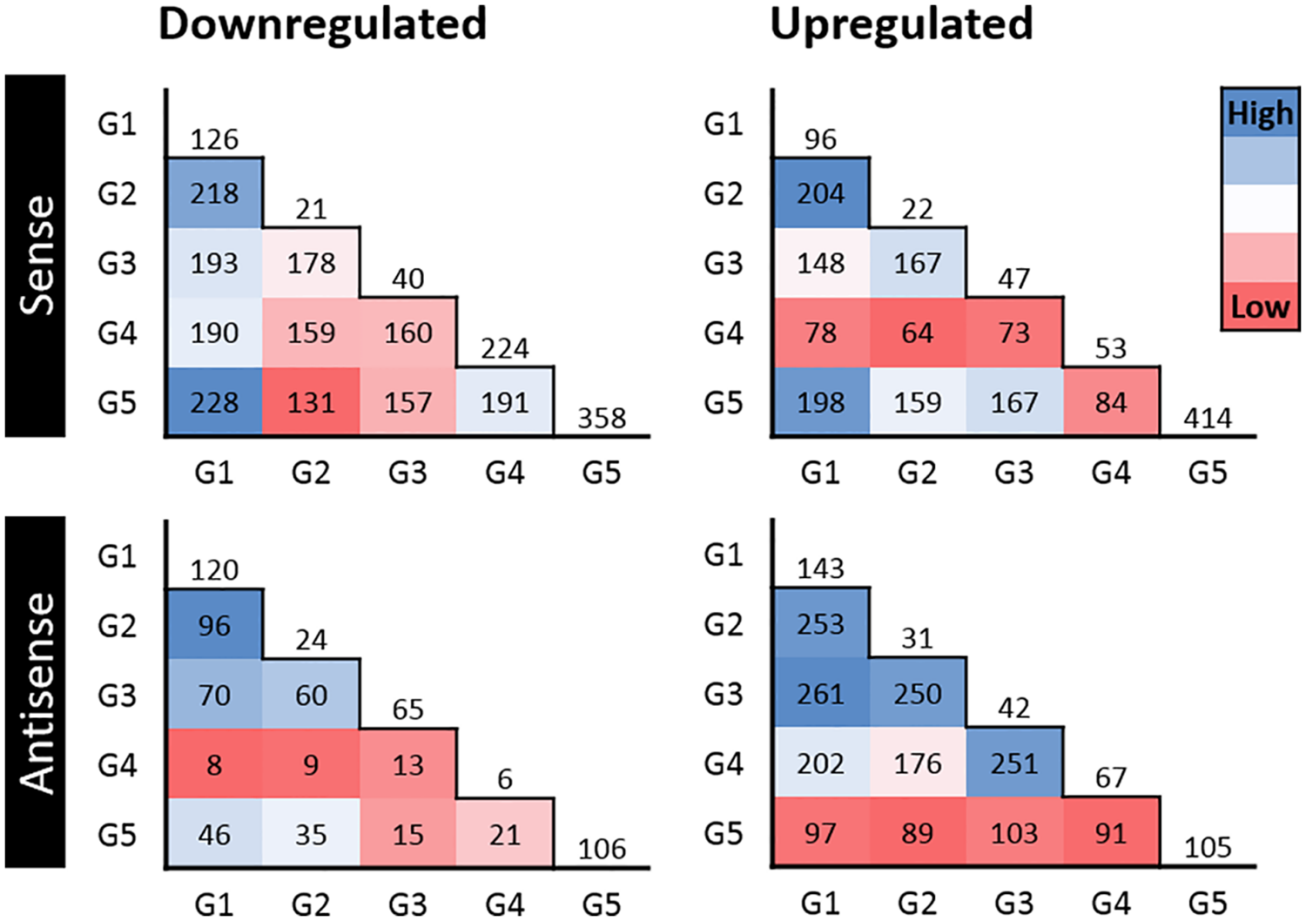
Heatmaps showing the number of *L*. *monocytogenes* sense and antisense RNA co-upregulated or co-downregulated (>2-fold) between pairs of growth phases at 4°C. Numbers outside of the pyramids represent the number of genes uniquely upregulated or downregulated at each growth phase. See Fig 1 for information about growth phases.

Fig 4 showed the pairs of growth phases that shared the highest and lowest numbers of the same up or downregulated genes at 4°C relative to 20°C. G4 had the lowest number of upregulated genes in common with the other growth phases, whereas G1 and G2 (G1:G2) and G1:G5 shared many of the same upregulated genes. With respect to downregulated genes, G4 and G5 had the smallest in common with G2 and G3 whereas G1:G2 and G1:G5 shared a high number of downregulated genes.

To confirm our DE findings, we used RT-qPCR to quantify the expression levels of a gene that was upregulated for >4 fold in response to cold at all growth phases (*cspB*), and a gene that was downregulated for >4 fold in response to cold at all growth phases (*leuA*), according to the RNA-seq data. We observed a strong positive correlation (R^2^ = 0.96, y = 1.20x-0.41) between the mRNA levels detected by the two methods. Nevertheless, the fold changes calculated using RNA-seq data were consistently larger than those obtained using RT-qPCR (S1 Fig).

### Core set of genes induced by cold stress

The DE analyses revealed a core set of 22 genes induced at 4°C relative to 20°C at all five growth phases (Table 2). These genes fall into the category of cold acclimation proteins (CAPs), which are defined as proteins encoded by genes that show continuous increased expression throughout prolonged cold-stress exposure [8, 50]. Among these CAPs were nine genes involved in the production of branched-chain amino acids (BCAAs) (*ilvDBHC-leuABCD-ilvA*). These BCAA synthesis genes were statistically overrepresented among the upregulated genes at all growth phases (Table 3). The BCAAs leucine, isoleucine, and valine, are critical for BCFA production, with isoleucine being the precursor for anteiso BCFAs. *Listeria* and similar bacteria, such as *Bacillus* species, alter their membrane lipid composition in response to temperature downshifts, to include higher percentages of branched-chain fatty acids (BCFAs) with anteiso configurations [24, 51–53]. Anteiso BCFAs have lower melting points than their iso-branched and straight-chain counterparts. They are thus more effective at increasing membrane fluidity, which is needed to maintain membrane-transport functions at low temperatures.

**Table 2:** Core set of genes upregulated (>2-fold) across multiple (≥4) growth phases in *L*. *monocytogenes* cells at 4°C vs. 20°C.

EGD ORF	EGD description	Gene abbr.	Strand	EGD-e ORF	Cluster memb-ership	Log_2_ DE across five growth phases				
						G1	G2	G3	G4	G5
*LMON_0187*	Veg protein		+	*lmo0189*	16/14	**2.96**	**2.45**	**2.28**	**2.38**	**1.54**
*LMON_0209*	LSU ribosomal protein L25p	*ctc*	+	*lmo0211*	10	**2.16**	**3.43**	**2.72**	**0.87**	**1.58**
*LMON_0260*	Internalin H	*inlH*	+	*lmo0263*	12	**2.21**	**3.67**	**2.11**	**-1.54**	**1.57**
*LMON_0261*	Internalin D	*inlD*	+		9	**1.86**	**2.79**	**2.18**	**1.23**	**2.70**
*LMON_0350*	Transaldolase			*lmo0343*	5	**1.88**	**2.03**	**1.56**	0.27	**4.16**
*LMON_0351*	3-oxoacyl-[acyl-carrier protein] reductase			*lmo0344*	5	**2.35**	**1.71**	**1.30**	0.62	**4.34**
*LMON_0352*	Ribose 5-phosphate isomerase B			*lmo0345*	5	**2.75**	**2.30**	**1.25**	0.26	**4.61**
*LMON_0353*	Triosephosphate isomerase			*lmo0346*	5	**2.38**	**1.95**	**1.75**	0.45	**4.38**
*LMON_0354*	Phosphoenolpyruvate-dihydroxyacetone phosphotransferase, ADP-binding subunit DhaL			*lmo0347*	5	**2.21**	**2.08**	**1.70**	0.03	**4.21**
*LMON_0355*	Phosphoenolpyruvate-dihydroxyacetone phosphotransferase, dihydroxyacetone binding subunit DhaK			*lmo0348*	5	**2.34**	**1.85**	**1.60**	0.26	**4.50**
*LMON_0356*	Hypothetical protein			*lmo0349*	5	**1.94**	**2.17**	**1.35**	-0.16	**5.37**
*LMON_0357*	Hypothetical protein			*lmo0350*	5	**3.01**	**1.62**	**1.45**	-0.25	**5.55**
*LMON_0358*	Phosphoenolpyruvate-dihydroxyacetone phosphotransferase, subunit DhaM			*lmo0351*	5	**2.49**	**1.79**	**1.57**	-0.37	**5.50**
*LMON_0360*	Acetyltransferase, GNAT family		+	*lmo0353*	12	**1.85**	**1.50**	**1.23**	0.67	**1.72**
*LMON_0441*	Internalin A	*inlA*	+	*lmo0433*	5	**2.01**	**1.82**	**1.92**	**1.62**	**2.30**
*LMON_0486*	LSU ribosomal protein L32p	*rpmF*	+	*lmo0486*	19	**1.43**	**1.46**	**1.76**	**1.75**	**1.22**
*LMON_0515*	Universal stress protein		+	*lmo0515*	12	**1.34**	**1.76**	**1.12**	**-2.06**	**1.98**
*LMON_0561*	Phosphoribosyl-ATP pyrophosphatase	*hisE*		*lmo0561*	2	**1.70**	**1.64**	**1.01**	**1.53**	**2.98**
*LMON_0611*	Internalin-like protein			*lmo0610*	12	**2.50**	**2.85**	**2.06**	-0.73	**2.19**
*LMON_0625*	Acetyltransferase, GNAT family			*lmo0624*	5	**2.07**	**2.19**	**1.67**	**1.33**	**4.46**
*LMON_0626*	Hypothetical protein			*lmo0625*	5	**2.18**	**2.19**	**1.59**	**1.23**	**4.43**
*LMON_0785*	PTS system, mannose-specific IID component			*lmo0781*	12	**1.81**	**2.76**	**1.32**	-0.54	**1.97**
*LMON_0786*	PTS system, mannose/fructose-specific IIC component			*lmo0782*	12	**1.60**	**2.72**	**1.43**	-0.76	**1.64**
*LMON_0787*	PTS system, mannose-specific IIB component			*lmo0783*	12	**1.86**	**2.80**	**1.43**	**-1.11**	**1.86**
*LMON_0788*	PTS system, mannose-specific IIB component / IIA component			*lmo0784*	12	**2.05**	**3.10**	**1.49**	**-1.23**	**1.70**
*LMON_0920*	Succinate-semialdehyde dehydrogenase [NAD]		+	*lmo0913*	10	**4.64**	**5.74**	**2.96**	0.40	**2.35**
*LMON_0944*	Hypothetical protein				10	**2.77**	**3.84**	**4.22**	**1.39**	**2.00**
*LMON_1488*	Osmotically activated L-carnitine/choline ABC transporter, permease protein OpuCD	*opuCD*		*lmo1425*	10	**2.34**	**5.37**	**3.52**	**1.79**	**1.29**
*LMON_1489*	Osmotically activated L-carnitine/choline ABC transporter, substrate-binding protein OpuCC	*opuCC*		*lmo1426*	10	**2.45**	**5.23**	**3.78**	**1.82**	**1.24**
*LMON_1490*	Osmotically activated L-carnitine/choline ABC transporter, permease protein OpuCB	*opuCB*		*lmo1427*	10	**2.51**	**5.28**	**3.78**	**1.58**	**1.10**
*LMON_1491*	Osmotically activated L-carnitine/choline ABC transporter, ATP-binding protein OpuCA	*opuCA*		*lmo1428*	10	**2.63**	**5.54**	**3.94**	**1.30**	-0.01
*LMON_1932*	Predicted membrane protein hemolysin III		+	*lmo1864*	14	**2.21**	**1.58**	**1.78**	**1.84**	**1.06**
*LMON_2054*	Dihydroxy-acid dehydratase	*ilvD*	+	*lmo1983*	4*	**3.52**	**3.89**	**3.34**	**4.29**	**3.62**
*LMON_2055*	Acetolactate synthase large subunit	*ilvB*	+	*lmo1984*	3/15	**3.67**	**4.17**	**3.41**	**4.41**	**4.37**
*LMON_2056*	Acetolactate synthase small subunit	*ilvH*	+	*lmo1985*	15/1	**3.67**	**4.02**	**3.39**	**4.41**	**4.03**
*LMON_2057*	Ketol-acid reductoisomerase	*ilvC*	+	*lmo1986*	4	**2.98**	**4.08**	**3.00**	**3.96**	**3.28**
*LMON_2058*	2-isopropylmalate synthase	*leuA*	+	*lmo1987*	11	**3.41**	**3.68**	**2.74**	**3.56**	**3.87**
*LMON_2059*	3-isopropylmalate dehydrogenase	*leuB*	+	*lmo1988*	3	**3.18**	**3.60**	**2.86**	**3.84**	**4.72**
*LMON_2060*	3-isopropylmalate dehydratase large subunit	*leuC*	+	*lmo1989*	3	**3.29**	**3.48**	**2.72**	**3.72**	**5.25**
*LMON_2061*	3-isopropylmalate dehydratase small subunit	*leuD*	+	*lmo1990*	3	**3.16**	**3.64**	**2.71**	**3.96**	**5.70**
*LMON_2062*	Threonine dehydratase	*ilvA*	+	*lmo1991*	3	**3.16**	**3.65**	**2.50**	**3.86**	**5.58**
*LMON_2234*	Hypothetical protein			*lmo2158*	12	**3.80**	**5.25**	**3.18**	0.77	**3.03**
*LMON_2407*	Internalin-like protein		+	*lmo2396*	3	**1.13**	**1.52**	**0.92**	**1.30**	**2.67**
*LMON_2696*	Universal stress protein		+	*lmo2673*	12	**3.09**	**3.64**	**1.90**	-0.77	**4.24**
*LMON_2718*	Phosphoenolpyruvate-dihydroxyacetone phosphotransferase, dihydroxyacetone binding subunit DhaK		+	*lmo2695*	12	**2.73**	**3.82**	**2.25**	-0.43	**2.36**
*LMON_2719*	Phosphoenolpyruvate-dihydroxyacetone phosphotransferase, ADP-binding subunit DhaL		+	*lmo2696*	12	**2.76**	**3.76**	**2.13**	0.31	**2.61**
*LMON_2720*	Phosphoenolpyruvate-dihydroxyacetone phosphotransferase, subunit DhaM;		+	*lmo2697*	12	**2.46**	**3.79**	**2.13**	0.58	**2.82**
*LMON_2735*	Secreted protein			*lmo2713*	1	**2.10**	**1.16**	**1.12**	**1.69**	**2.03**
*LMON_2770*	general stress protein 26			*lmo2748*	12	**2.51**	**3.72**	**1.71**	**-1.81**	**1.98**
*LMON_2800*	PTS system, cellobiose-specific IIA component			*lmo2780*	12	**1.79**	**1.57**	**1.45**	-0.55	**2.57**
*LMON_2801*	Beta-glucosidase			*lmo2781*	12	**2.06**	**1.38**	**1.45**	-1.18	**2.27**
*LMON_2802*	PTS system, cellobiose-specific IIB component			*lmo2782*	12	**2.11**	**1.49**	**1.81**	-1.48	**2.44**
*LMON_2803*	PTS system, cellobiose-specific IIC component			*lmo2783*	12	**2.32**	**1.24**	**1.80**	-1.99	**2.05**

* indicates <0.50 cluster membership; G1–G5 refer to differential expression in *L*. *monocytogenes* cells grown at 4°C relative to 20°C and across five specific growth phases (see Fig 1); Blue shading indicates genes with significantly increased (> 1log_2_, p<0.05) gene expression and yellow shading indicates genes with significantly decreased (< -1log_2_, p<0.05) expression at 4°C relative to 20°C; Bolded values indicate significant (p<0.05) differential expression changes.

**Table 3:** Pathways and gene ontology processes significantly (p<0.05*) enriched among genes upregulated (>2-fold) at 4°C vs. 20°C.

Biological process or pathway	Gene examples	p-value (# of contributing genes)				
		G1	G2	G3	G4	G5
Branched-chain amino acid synthesis	*ilvABCDH*, *leuABCD*,	1.18E-6 (9)	8.04E-8 (9)	9.14E-8 (9)	7.46E-11 (9)	5.75E-4 (9)
Arginine biosynthesis	*argBDGHJ*, *carbB*	3.42E-2 (4)	4.77E-4 (6)	4.25E-5 (7)		4.37E-3(8)
Glycerol degradation	*LMON_0354–355*, *0358*, *2718–2720*	2.87E-2 (6)	1.41E-5 (8)	9.82E-7 (9)		4.06E-3 (9)
Histidine biosynthesis	*hisABDEGIZ*	5.58E-5 (9)	7.20E-3 (4)		5.99E-9 (7)	1.16E-2 (6)
Methionine biosynthesis	*metX*, *LMON_0595*, *0847*		4.99E-3 (3)		3.57E-2 (3)	
Oxidation-reduction process (GO:0055114)	*qoxABCD*, *gabD*		4.37E-2 (4)	1.54E-6 (8)		2.46E-4 (9)
Response to stress (GO:0006950)	*recF*, *hrcA*, *grpE*, *dnaK*, *ltrC*, *uspA*, *csbD*, *ctsR*, *clpP*, *clpE*	1.96E-5 (13)				
Biological regulation (GO:0065007)	*yycF*, *ctsR*, *hrcA*, *frvA*, *opuCD*	2.89E-3 (10)				
Pyrimidine ribonucleotide biosynthesis	*pyrABC*, *carB*				1.24E-3 (4)	
Pentose phosphate pathway	*LMON_2682–2685*, *2697*, *0349*, *0350*, *0352*					1.23E-3 (13)
Carboxylates degradation	*fruA*, *bvrB*, *LMON_0095–0097*, *2706–2708*, *0785–0788*					7.60E-6 (44)
Tryptophan biosynthesis	*trpBCDFG*)					2.96E-2 (5)
Purine ribonucleotide biosynthesis	*purMQDFN*					2.96E-2 (5)
Active transporter activity (GO:0022804)	*opuCD*, *mpoABCD*, *mptACD*, PTS systems					1.56E-8 (42)
Structural constituent of ribosome (GO:0003735)	*rplEFNOPVX*, *rpsEHNP*, *rpmC*					2.90E-2 (20)

* Statistical overrepresentation of gene sets were determined using Fisher’s exact test and the BioCyc database.

Other genes with increased expression in response to cold stress at all growth phases, included those encoding the L-carnitine transporter OpuC (*opuCB-opuCC-opuCD*), internalins A and D, a ribosomal protein (RpmF), a histidine biosynthesis protein (HisE), a GNAT family acetyltransferase (*LMON*_*0625*), and Veg protein (*LMON*_*0187*). The roles of carnitine and ribosomal proteins in bacterial adaptation to certain stresses have been previously reported [18, 54–56]. In *B*. *subtilis* Veg protein has been shown to activate extracellular matrix genes and contribute to biofilm formation [57]. In *L*. *monocytogenes*, increased expression of Veg has been observed following exposure to acid and cold stress [11, 58], but its function remains unknown.

In addition to the upregulation of *inlA* and *inlD* at all growth phases at 4°C, *inlH* and two genes encoding internalin-like proteins (*LMON*_*2407*, *LMON_0611*) were upregulated at four growth phases (Table 2). The most recognized roles of internalins A and B are in *L*. *monocytogenes*’ virulence. Nevertheless, the *Listeria* spp. family of internalin proteins all share a leucine-rich-repeat domain that allows them to bind structurally unrelated ligands, thereby implicating them in a wide range of functions [59]. Recent studies have shown that isolates containing a full-length version of *inlA* exhibit enhanced cold tolerance relative to those with a truncated version [39, 60]. Further research is needed to confirm the potential role(s) of internalins in the *L*. *monocytogenes* CSR.

**Fig 5:**
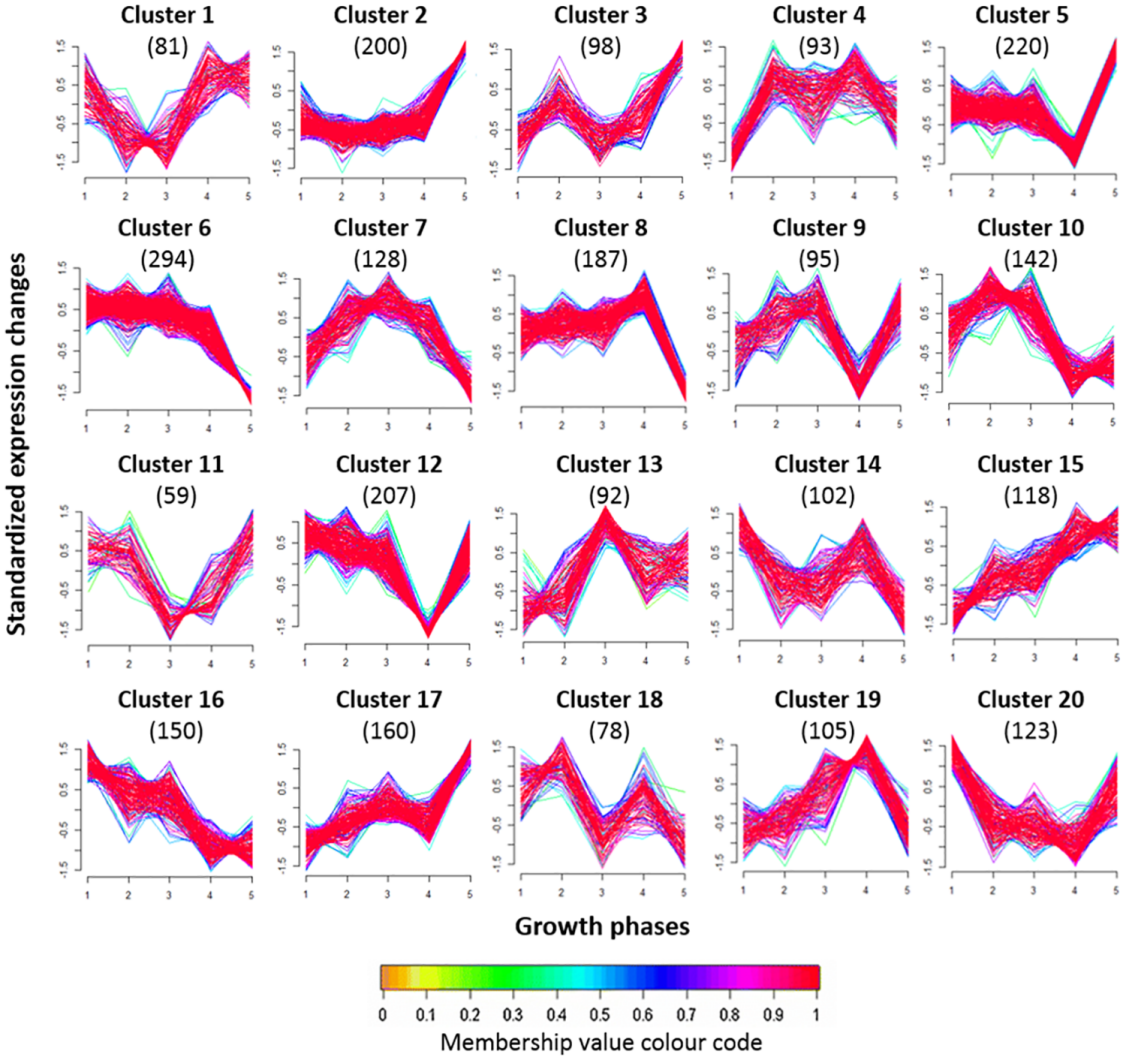
Differential gene expression patterns observed in *L*. *monocytogenes* cells grown across five growth phases at 4°C (see Fig 1). Clusters were formed using fuzzy c-means soft clustering and standardized log_2_ fold change values for 4°C expression levels vs. 20°C expression levels at each growth phase. Numbers in parentheses denote the number of genes in each cluster core. Represented genes had cluster membership values >0.5. See Fig 1 for information about growth phases.

Many more genes (n = 104) were upregulated at four growth phases at 4°C relative to 20°C, with the majority being induced at G1, G2, G3, and G5 (G1–G3:G5, n = 70). These genes shared similar DE patterns across all growth phases (Fig 5. Cluster 5 and 12), where levels of DE dramatically decrease at G4. Present in this group were genes involved in arginine biosynthesis (*dapE*, *argGHJ*), general stress response (*LMON*_*0515*, *LMON_2234*, *LMON_2696*, *LMON_2770*), FA synthesis (*LMON*_*0351*), and phosphotransferase system (PTS) uptake of cellobiose (*LMON*_*2800–2803*) and mannose (*LMON*_*0785–0788*) (Table 2). Additionally, the genes encoding succinate-semialdehyde dehydrogenase (*LMON*_0920), 11 genes from the pentose phosphate pathway (*LMON*_0350–0360), and two sets of genes (*LMON*_*0354–0356*, *LMON_2718–2720*) encoding dihydroxyacetone kinase (DhaKLM) were upregulated in these growth phases (Table 2).

An additional 100 genes displayed induced transcription at three growth phases, with the largest number co-upregulated at G1–G2:G5 (n = 29), followed by G1–G3 (n = 24). The similarities between these sets of growth phases are visible in Fig 4. At G1–G2:G5, *L*. *monocytogenes* growth is inhibited, and such conditions would be expected to induce genes encode proteins associated with 1) secondary membrane-transport systems that are less energy demanding, 2) the utilization of alternative energy sources, and 3) cell detoxification. It appears that the demand for these proteins is further increased in cold stressed cells as we observed the upregulation of genes involved in utilizing ethanolamine as an alternative energy source (*LMON*_*1167–1180*), oligopeptides transport (*oppC*), transcription regulation (5 genes), general stress response (*clpC*), and ribosomal subunit assembly (*LMON*_*2523*). At G1–G3, several of the shared upregulated genes encoded reductases (*LMON_1496*, *LMON_2403*, *LMON_0798*, *LMON_0643*, *LMON_2843*) with known or putative roles in maintaining redox balance within the cell and managing oxidative stress.

### Core set of genes suppressed under cold stress

A total of 42 genes were downregulated at 4°C in all growth phases, including genes involved in cold shock (*cspB*), virulence (*actA*, *plcB*, *capA*, *mpl*), nucleotide degradation (*LMON*_*0129*, *LMON*_*1234*, *guaB*), vitamin B9 and B12 synthesis (*LMON*_*1163*, *LMON_2771–2772*), glycerophospholipid export (*LMON*_*0106–0107*), and sugar uptake (*LMON*_*0770–0772*, *LMON*_*1451–1452*). Many of the genes suppressed at 4°C contributed to either cobalamin (B12) biosynthesis and subsequent utilization, or the production and export of cell wall components (Table 4).

**Table 4:** Pathways and gene ontology processes significantly (p<0.05*) enriched among genes downregulated (>2-fold) at 4°C vs. 20°C.

Biological process or pathway	Gene examples	p-value (# of contributing genes)				
		G1	G2	G3	G4	G5
Cobalamin biosynthesis	*cbiKA*, *cbiP*	5.82E-4 (5)		5.14E-6 (6)	4.01E-3 (6)	4.33E-4 (5)
Biosynthesis of cell wall macrostructures (GO:0070589)	*dltABCD*, *pgdA*, *murABCE*, *pgi*, *tagD*	5.76E-4 (6)	3.94E-4 (5)	1.67E-3 (5)		1.02E-4 (20)
Pathogenesis	*inlK*, *inlJ*, *actA*, *arcB*, *flaA*, *ctaP*	1.13E-2 (9)	4.83E-3 (7)	2.62E-2 (7)		
Pyrimidine nucleotides/UMP biosynthesis	*pyrCBFGHR*, *comEB*, *udk*, *carB*	2.60E-3 (8)	1.64E-6 (9)	3.03E-6 (11)		
Localization (GO:0051179)	*dtpT*, *phoU*, *pstB*, *ctaP*	1.03E-2 (13)	2.52E-3 (10)		4.59E-2 (12)	
Transport (GO:006810)	*ctpA*, *pstB*, *phoU*	1.03E-2 (13)	2.52E-3 (10)		4.59E-2 (12)	
Carboxylate degradation	*PdhABC*		1.11E-4 (21)		3.92E-8 (43)	
UDP biosynthesis	*murABCE*, *racE*, *ddl*	3.32E-3 (6)				2.74E-2 (6)
tRNA charging	*metS*, *pheS*, *serS*, *tyrS*, *aspS*, *alaS*, *argS*	1.80E-2 (8)				
Myo-inositol degradation	*LMON_2235*, *2238–2239*				1.54E-2 (4)	
Nucleotide degradation	*pdp*, *dra*, *drm*, *pnp*, *guaB*				1.29E-2 (10)	
Biosynthesis	*folD*, *cbiP*, *gatAC*, *comEB*, *hemABCD*, *cycEK*, *tagD*, *cdsA*					5.50E-5 (128)
Cofactors and electron carrier biosynthesis	*hemABCD*, *menEF*, *nadE*, *cbiP*, *ubiE*					2.20E-3 (40)

* Statistical overrepresentation of gene sets were determined using Fisher’s exact test and the BioCyc database.

Cobalamin derivatives function as essential cofactors for important enzymes that catalyze a variety of transmethylation and rearrangement reactions [61]. In bacteria, cobalamin is necessary to degrade ethanolamine, propanediol, glycerol, and pyruvate and to produce carbon and energy [61–63]. *L*. *monocytogenes* and other bacteria induce the transcription of both cobalamin biosynthesis and ethanolamine utilization genes under a variety of stress conditions [64–68]. However, in the present study, we observed the downregulation of cobalamin biosynthesis genes and others encoding cobalamin-dependent enzymes and pathways (*eutB*, *LMON*_*1144–1154*, *LMON*_*1803*, *LMON_0942*), whereas the ethanolamine utilization operon remained upregulated at several growth phases.

Genes involved in the biosynthesis and export of peptidoglycan, teichoic acids, cell wall proteins, and membrane lipids were also suppressed at 4°C (Table 4). Both peptidoglycan and teichoic acid biosynthesis stem from the precursor molecule UDP-N-acetyl-α-D-glucosamine (UDP-GlcNAc) and require the attachment of D-alanine residues, and for peptidoglycan also glutamate. Correspondingly, genes involved in UDP-GlcNAc biosynthesis and transport, alanine attachment (*dltABCD*), and glutamate transport (*LMON*_*0849–0850*) and attachment (*racE*) were downregulated. Other suppressed genes included several fatty-acid-coA ligases, lipid export proteins, and enoyl-acyl-carrier proteins and reductases, which collectively assist in the production and export of phospholipids, cell wall-associated lipoproteins, and lipoteichoic acids. These findings suggest a reduced rate of peptidoglycan/cell envelope turnover at 4°C relative to 20°C. This reduced turnover rate may reflect the reduced cellular growth rate at this temperature, or conservation of carbohydrates for alternative uses.

Other genes suppressed at 4°C had roles in localization and pathogenicity, heme biosynthesis, tRNA processing and charging, nucleotide degradation and salvage, and carbohydrate transport and catabolism (Table 4). Not surprising, many of these genes also have alternative roles in cell wall biogenesis. Notably, over 50 genes from PTS operons were downregulated at one or more growth phases, far exceeding the number of PTS genes upregulated in response to cold stress. PTSs utilize phosphate to facilitate the uptake of simple sugars and thus consume more energy than other membrane kinases with the same sugar specificity [69]. This suggests that *L*. *monocytogenes* may benefit from employing alternative sugar uptake systems when exposed to cold stress to conserve energy for more critical CSR mechanisms.

### *L*. *monocytogenes* cold-stress response at individual growth phases

#### Early lag phase (G1)

Previous studies have described the lag phase following cold stress as the acclimatization phase. In this phase, bacteria suppress bulk protein synthesis, and dramatically increase production of transient cold induced stress response proteins (CIPs) [8]. Once the cells are cold adapted, CIP synthesis ends and bulk protein synthesis and cell growth resume. Depending on the degree of cold stress, the types and expression levels of CIPs will differ. Some of the functions associated with CIPs include DNA unwinding, nucleic acid stabilization, and compatible solute uptake [70, 71].

A total of 96 genes were uniquely induced at G1, accounting for 25% of the genes upregulated at this growth phase. Most of these genes had DE patterns that belonged to clusters 14, 16, or 20 (Fig 5), and a large number encoded transcription regulators (n = 19), including HrcA, CtsR, DegU, and YycF. Both HrcA and CtsR are negative regulators of several heat-shock and general stress-response genes, a number of which were also upregulated at G1 (*grpE*, *dnaK*, *clpB*, *gmpA*, *mscAB*, *clpC*, *clpE*, *and clpP*). Many of these upregulated genes, including *groEL* and *groES*, encode chaperone proteases capable of degrading the misfolded or damaged proteins that would probably occur following a rapid temperature downshift. By contrast, the transcription regulator DegU contributes to motility, growth at high temperatures, and efficient biofilm formation [72]. Co-transcribed with *degU* is *yviA*, which encodes a DegV family protein with putative functions in FA transport or metabolism [72, 73]. As we did not observe upregulation of genes known to be activated by DegU, the role of this operon in early cold adaptation may be primarily associated with the functions of YviA. In support of this hypothesis, YycF, a transcription regulator of FA biosynthesis and cell wall metabolism genes [74], was also upregulated at G1.

Additional genes upregulated at G1 encode proteins involved in DNA repair (*recF*, *gyrAB*, *recX*, *LMON*_*0230*, *LMON_1225*), ethanolamine utilization (*eutABCM*, *LMON*_*1166–1180*), carnitine uptake (*opuCABCD*), phosphate transport and metabolism (*pstABCS*, *phoU*, *phoB*), RNA stability (*LMON*_*0869*, *LMON_1037*), nitrogen utilization (*LMON*_*1582–1583*), and histidine and arginine biosynthesis. Two genes with >20-fold increased expression at G1 encode succinate-semialdehyde dehydrogenase [NAD] (*LMON*_*0920*) and pyruvate phosphate dikinase (*LMON*_*1936*) (Table 5). These proteins function in energy production, and glycerol metabolism, respectively.

**Table 5:** Top 10 most highly induced genes in *L*. *monocytogenes* cells at 4°C vs 20°C at five different growth phases.

EGD ORF	EGD description	Gene abbr.	Strand	EGD-e ORF	Cluster memb-ership	Log_2_ DE across five growth phases				
						G1	G2	G3	G4	G5
**G1 –early lag phase**										
*LMON_0762*	Hypothetical protein		+	*lmo0758*	20	**3.53**	-0.35	0.48	**-1.47**	**1.75**
*LMON_0763*	glyoxalase family protein		+	*lmo0759*	20	**3.55**	0.04	**1.28**	-0.61	**1.78**
*LMON_0765*	Nitrilotriacetate monooxygenase component B		+	*lmo0761*	20	**3.66**	**0.53**	**1.49**	0.70	**1.99**
*LMON_0920*	Succinate-semialdehyde dehydrogenase [NAD]		+	*lmo0913*	10	**4.64**	**5.74**	**2.96**	0.40	**2.35**
*LMON_1761*	Hypothetical protein		+	*lmo1694*	10	**3.78**	**5.06**	**2.55**	-0.95	**0.67**
*LMON_1936*	Pyruvate,phosphate dikinase		+	*lmo1867*	20	**4.31**	**2.17**	**1.90**	0.47	**2.16**
*LMON_2054*	Dihydroxy-acid dehydratase	*ilvD*	+	*lmo1983*	4*	**3.52**	**3.89**	**3.34**	**4.29**	**3.62**
*LMON_2055*	Acetolactate synthase large subunit	*ilvB*	+	*lmo1984*	3/15	**3.67**	**4.17**	**3.41**	**4.41**	**4.37**
*LMON_2056*	Acetolactate synthase small subunit	*ilvH*	+	*lmo1985*	15/1	**3.67**	**4.02**	**3.39**	**4.41**	**4.03**
*LMON_2234*	Hypothetical protein			*lmo2158*	12	**3.80**	**5.25**	**3.18**	0.77	**3.03**
**G2 –transition to exponential growth phase**										
*LMON_0020*	Hypothetical protein			*lmo0019*	10	**2.62**	**5.25**	**3.85**	0.65	**1.64**
*LMON_0920*	Succinate-semialdehyde dehydrogenase [NAD]		+	*lmo0913*	10	**4.64**	**5.74**	**2.96**	0.40	**2.35**
*LMON_1003*	Hypothetical protein			*lmo0994*	12	**3.09**	**5.04**	**2.48**	-0.56	**2.59**
*LMON_1488*	L-carnitine/choline ABC transporter, permease protein	*opuCD*		*lmo1425*	10	**2.34**	**5.37**	**3.52**	**1.79**	**1.29**
*LMON_1489*	L-carnitine/choline ABC transporter, substrate-binding protein	*opuCC*		*lmo1426*	10	**2.45**	**5.23**	**3.78**	**1.82**	**1.24**
*LMON_1490*	L-carnitine/choline ABC transporter, permease protein	*opuCB*		*lmo1427*	10	**2.51**	**5.28**	**3.78**	**1.58**	**1.10**
*LMON_1491*	L-carnitine/choline ABC transporter, ATP-binding protein	*opuCA*		*lmo1428*	10	**2.63**	**5.54**	**3.94**	**1.30**	-0.01
*LMON_1761*	Hypothetical protein		+	*lmo1694*	10	**3.78**	**5.06**	**2.55**	-0.95	**0.67**
*LMON_2234*	Hypothetical protein			*lmo2158*	12	**3.80**	**5.25**	**3.18**	0.77	**3.03**
*LMON_2306*	Arsenate reductase		+	*lmo2230*	10	**3.36**	**4.77**	**2.95**	**-1.12**	**1.49**
**G3 –mid-exponential growth phase**										
*LMON_0369*	Twin-arginine translocation protein	*tatC*		*lmo0361*	13	-0.08	**0.57**	**4.35**	0.25	**3.49**
*LMON_0370*	Twin-arginine translocation protein	*tatA*		*lmo0362*	13	-0.06	**0.94**	**5.30**	0.65	**3.26**
*LMON_0373*	Ferrous iron transport permease	*efeU*	+	*lmo0365*	13	-0.66	**0.87**	**4.80**	**2.03**	**3.72**
*LMON_0374*	Ferrous iron transport periplasmic protein	*efeO*	+	*lmo0366*	13	**-1.18**	**0.87**	**4.60**	**1.12**	**3.74**
*LMON_0375*	Ferrous iron transport peroxidase	*efeB*	+	*lmo0367*	13	**-1.08**	**1.04**	**4.55**	**1.33**	**3.74**
*LMON_0541*	ABC transporter, substrate-binding protein			*lmo0541*	13	**-0.95**	-0.08	**4.34**	0.95	**2.00**
*LMON_0944*	Hypothetical protein				10	**2.77**	**3.84**	**4.22**	**1.39**	**2.00**
*LMON_1016*	Hypothetical protein		+	*lmo1007*	13	0.17	**1.14**	**4.89**	**1.41**	**3.19**
*LMON_2261*	Cell surface protein IsdA, transfers heme from hemoglobin to apo-IsdC	*isdA*		*lmo2185*	13	**-1.77**	-0.61	**4.21**	0.36	**3.24**
*LMON_2262*	NPQTN cell wall anchored protein	*isdC*		*lmo2186*	13	**-1.50**	**-0.56**	**4.38**	0.95	**4.24**
**G4 –transition to stationary phase**										
*LMON_1312*	Membrane protein			*lmo1252*	19/15	-0.07	0.15	-0.01	**4.16**	**1.28**
*LMON_2054*	Dihydroxy-acid dehydratase	*ilvD*	+	*lmo1983*	4*	**3.52**	**3.89**	**3.34**	**4.29**	**3.62**
*LMON_2055*	Acetolactate synthase large subunit	*ilvB*	+	*lmo1984*	3/15	**3.67**	**4.17**	**3.41**	**4.41**	**4.37**
*LMON_2056*	Acetolactate synthase small subunit	*ilvH*	+	*lmo1985*	15/1	**3.67**	**4.02**	**3.39**	**4.41**	**4.03**
*LMON_2057*	Ketol-acid reductoisomerase	*ilvC*	+	*lmo1986*	4	**2.98**	**4.08**	**3.00**	**3.96**	**3.28**
*LMON_2059*	3-isopropylmalate dehydrogenase	*leuB*	+	*lmo1988*	3	**3.18**	**3.60**	**2.86**	**3.84**	**4.72**
*LMON_2061*	3-isopropylmalate dehydratase small subunit	*leuD*	+	*lmo1990*	3	**3.16**	**3.64**	**2.71**	**3.96**	**5.70**
*LMON_2062*	Threonine dehydratase	*ilvA*	+	*lmo1991*	3	**3.16**	**3.65**	**2.50**	**3.86**	**5.58**
*LMON_2286*	Hypothetical protein			*lmo2210*	19	**1.59**	**0.71**	**2.28**	**3.79**	**2.33**
*LMON_2534*	Cell wall-binding protein			*lmo2522*	4	**-1.33**	**2.20**	**0.95**	**4.50**	**1.70**
**G5 –late-stationary phase**										
*LMON_0740*	D-allulose-6-phosphate 3-epimerase		+	*lmo0735*	2	-0.81	**-1.52**	**-2.11**	-1.19	**10.05**
*LMON_0741*	D-allose-6-phosphate isomerase		+	*lmo0736*	2	-0.52	**-1.36**	**-1.52**	-0.35	**10.41**
*LMON_0742*	Hypothetical protein		+	*lmo0737*	2	0.04	**-0.98**	**-1.86**	1.08	**10.49**
*LMON_0743*	PTS system, D-allose-specific		+	*lmo0738*	2	**-0.61**	-0.29	**-0.59**	**1.52**	**9.56**
*LMON_0744*	6-phospho-beta-glucosidase		+	*lmo0739*	2	**-0.85**	-0.15	**-0.41**	0.86	**8.78**
*LMON_2673*	PTS system, lactose/cellobiose specific IIB subunit			*lmo2650*	2	**-2.19**	**-3.61**	**-1.53**	**-1.74**	**8.51**
*LMON_2674*	PTS system, IIA component			*lmo2651*	2	**-2.41**	**-4.12**	**-1.54**	**-1.80**	**9.19**
*LMON_2706*	PTS system, cellobiose-specific IIB component		+	*lmo2683*	17	**-1.46**	**-1.30**	**1.32**	-2.04	**10.66**
*LMON_2707*	PTS system, cellobiose-specific IIC component		+	*lmo2684*	17	**-1.57**	**-1.40**	**1.44**	**-2.83**	**9.36**
*LMON_2708*	PTS system, beta-glucoside/cellobiose-specific IIA component		+	*lmo2685*	17	**-1.38**	**-1.28**	**1.33**	**-2.43**	**9.28**

* indicates <0.50 cluster membership; G1–G5 refer to differential expression in *L*. *monocytogenes* cells grown at 4°C relative to 20°C and across five specific growth phases (see Fig 1); Blue shading indicates genes with significantly increased gene expression (> 1 log_2_, p<0.05) and yellow shading indicates genes with significantly decreased expression (< -1 log_2_, p<0.05) at 4°C relative to 20°C; Bolded values indicate significant (p<0.05) differential expression changes. Genes shaded in yellow are highly expressed at more than one growth phase.

Twenty-nine genes were solely induced at G1 and G2, highlighting their importance in the earlier CSR stages of *L*. *monocytogenes*. Included among these genes were those encoding the low temperature requirement protein LtrC, cold-shock protein CspL, regulatory protein RecX, and several proteins associated with phosphate transport (PstCABB-PhoU-PstS).

Forty-seven genes were upregulated at both G1 and G5 (late-stationary phase), many of which followed the DE pattern shown in cluster 16 (Fig 5). As discussed, these growth phases both represent stages in cold stress survival, where the population size is static and their induced genes are probably specific to surviving adverse conditions. Among these genes were those encoding heat-shock proteins (*clpP*, *clpE*, *groEL*, *LMON*_*2710–2711*), DNA gyrase (*gyrA*), an SOS-response protein (*lexA*), an RNA helicase (*secA*), and seven transcription regulators. Interestingly, although these genes were upregulated in response to cold stress in this study, several of them were also reported by van der Veen et al. [75] as induced in *L*. *monocytogenes* following 3 min of heat stress (48°C). As both stresses are caused by a rapid change temperature which causes molecular structures to take on different conformations; it makes sense they would share similarly upregulated genes with functions in DNA and RNA repair (*gyrA*, *lexA*, *secA*), and protein degradation (*clpP*, *clpE*) or stabilization (*groEL*).

#### Transition to exponential growth phase (G2)

At T2, cells have had an adequate amount of time to repair cold-induced damage to molecular structures, and to reprogram their cellular machinery to support growth at low temperatures. As T2 shares characteristics with both the lag and exponential phases, only 22 genes were uniquely upregulated at G2, and only 21 genes were uniquely downregulated. The exclusively induced genes encoded the iron binding protein, Fri; a cold-shock protein, CspD; and a DNA repair and SOS response protein, RecA. Most of these genes exhibited slightly less than a 2-fold increase in expression at G1, maximum increased expression at G2, and no change in expression or decreased expression from G3–G5, as is characteristic of cluster 10 (Fig 5). The upregulation of *fri*, *cspD*, and *recA* have been previously reported in *L*. *monocytogenes* in response to cold stress [16, 26, 27, 50].

Among the genes upregulated at G2, there was a significant (p<0.05) overrepresentation of those involved in glycerol metabolism and the synthesis of arginine, histidine, and BCAAs (Table 3). Among the most induced genes (>36 fold) were those encoding the OpuC carnitine uptake system (*opuCABCD*), hypothetical proteins, and succinate-semialdehyde dehydrogenase (*LMON_0920*) which was also strongly induced at G1 (Table 5).

#### Mid-exponential growth phase (G3)

A total of 47 genes were uniquely induced during exponential growth at 4°C, with most genes exhibiting the DE pattern shown in cluster 13 (Fig 5). Aside from a few genes with roles in iron transport (*fhuC*, *fhuB*, *LMON*_*2440–2441*) and oxidoreductase activity (*LMON*_*0560*, *LMON_2031*, *pdhD*), most are not known to share any common functions. Among G3-upregulated genes, there was an overrepresentation (p<0.05) of genes involved in glycerol metabolism, aerobic respiration, iron transport, and the biosynthesis of arginine, histidine, and BCAAs (Table 3). Other upregulated genes included those involved in the pentose phosphate pathway (*LMON*_*0349–0352*, *LMON_0647*), osmolyte uptake (*opuCABCD*), and 12 genes fell within PTS operons that facilitate the uptake of mannose, cellobiose, and beta-glucoside. Genes highly induced at G3 (>16-fold) were associated with iron (*efeUOB*) and heme transport (*isdCAEF*), as well as protein export (*tatAC*) (Table 5). As expected, many of the proteins previously induced during cold acclimatization (G1–G2) were downregulated during exponential growth.

#### Transition to stationary phase (G4)

Upon entry into stationary phase, cold-adapted cells downregulated almost 3× more genes than they upregulated (Fig 3). Unique to G4 was the upregulation of 53 genes with roles in glycine betaine transport (*gbuAB*), tRNA processing (*LMON*_*T39*, *T60*, *T66*), nucleotide biosynthesis (*pyrP*, *pyrB*, *pyrAa*, *pyrR*, *LMON_1838*), and maintaining a rod cell shape (*mreBD*), among others. Most of these genes displayed DE patterns that belonged to clusters 8 or 19 (Fig 5), in which DE peaks at G4 and falls dramatically at G5. The five most strongly induced genes (>16-fold) at this growth phase encoded three isoleucine biosynthesis proteins (*ilvDBH*) and two cell wall-associated proteins (*LMON_2534*, *LMON_1312*) (Table 5).

G4 and G5 also shared a set of exclusively upregulated genes (n = 23) with functions in nucleotide biosynthesis (*purAHNFE*, *pyrDII*, *carB*, *pyrC*), tRNA processing (*LMON*_*T51*), and cell-wall recycling (*LMON*_*0029*, *LMON_2776*), among others. All genes exhibited patterns of DE that belonged to cluster 1 or 15 (Fig 5). Nucleotide biosynthesis genes are likely activated at these growth phases to accommodate the increased demand for ribonucleic acids imposed by the large abundance of strongly upregulated genes at G5.

#### Late-stationary phase (G5)

Cold-adapted stationary-phase cells exhibited both the largest number of DE expressed genes and the largest magnitude of expression changes (Fig 3). Similar results were noted by Chan et al. [11] in their L. monocytogenes microarray study, which compared the cold-stress regulons of exponential and stationary-phase cells. More than 50% of G5-upregulated genes exhibited >4-fold increased expression and 17 genes, predominantly associated with PTS mediated sugar uptake systems, were upregulated between 100- and >1,000-fold. Many of the genes strongly induced by cold stress at this growth phase, also exhibited some of the highest expression levels in stationary-phase cells at 20°C, highlighting their overall importance during this growth phase and their enhanced importance in cold-adapted stationary-phase cells. Additionally, 50–60% of G5 genes were exclusively DE at this growth phase. The exclusively induced genes had roles in tryptophan (trpBCDFG, aroE) and ATP synthesis (atpACDFGH, adk, LMON_0091), creatine and rhamnose degradation, and ribosome and phage-related processes. Other genes encoded motility-associated proteins (motB, cheV, flaA), an osmosensitive K+ channel histidine kinase (kdpCDE), an Na(+) H(+) antiporter (LMON_2393–2396), the transcription repressor CodY, and low temperature requirement protein B (ltrB). Many genes associated with transcription and translation were also strongly induced at G5 (polC, dnaC, dnaI, topB, rbfA, mfd), including 28 transcription regulators, and 14 genes involved in tRNA processing (S1 Table).

Biological processes enriched (p<0.05) among the genes induced at G5 included arginine, histidine, ribonucleotide, and BCAA biosynthesis genes, as well as genes associated with carbohydrate uptake, the pentose phosphate pathway, glycerol and peptidoglycan degradation, and aerobic respiration (Table 3). The DE patterns shown in clusters 1, 2, 3, 5, 9, 11, 12, 15, 17, and 20 all describe genes with increased expression in cold-adapted stationary-phase cells, while clusters 4, 6–8, 10, 14, 16, 18, and 19 describe genes with limited or no roles in stationary-phase viability at low temperatures (Fig 5).

### *L*. *monocytogenes* cold stress regulon: A comparison with previous studies

Numerous previous studies have elucidated a large pool of genes with putative or known roles in the *L*. *monocytogenes* CSR [11–13, 16, 20, 27]. In this section, we will determine whether these genes are expressed in a cold-tolerant strain, whether a significant induction occurs when the downshift is from 20°C to 4°C (rather than from 37°C, as in most prior studies), and whether any growth-phase dependencies exist.

#### Osmolyte and oligopeptide uptake

In both L. monocytogenes and B. subtilis, low temperature and high osmolarity stress induce the intracellular accumulation of solutes and short peptides. These solutes and peptides function as osmoprotectants that facilitate cell growth under stressful conditions [18, 26, 54, 76–79]. In L. monocytogenes, carnitine and glycine betaine are the predominant solutes that accumulate [78–80], and their transport systems are encoded by the opuCABCD and gbuABC operons, respectively. Chan et al. [11] reported induction of opuCABCD and gbuC in cold-adapted L. monocytogenes exponential- but not stationary-phase cells, while Durack et al. [12] did not find that either system was induced in late exponential early stationary-phase cells. In the present study, opuCBCD was upregulated in all growth phases, while opuCA was upregulated at G1–G4 (Table 6). Regardless, all genes in the OpuC operon belonged to the DE pattern shown in cluster 10 (Fig 5), exhibiting the highest level of induction at G2 (up to 48-fold) and the lowest level at G5, which agrees with previous work [11, 12]. Similar to the findings of Chan et al. [11] gbuA and gbuB were only upregulated at G4 (Table 6).

**Table 6:** Genes commonly associated with the *L*. *monocytogenes* CSR.

EGD ORF	EGD description	Gene abbr.	Strand	EGD-e ORF	Cluster memb-ership	Log_2_ DE across five growth phases				
						G1	G2	G3	G4	G5
**Osmolyte and oligopeptide uptake**										
*LMON_1024*	Glycine betaine ABC transport system, ATP-binding protein	*gbuA*	+	*lmo1014*	8	0.15	**0.56**	**0.82**	**1.68**	**-0.69**
*LMON_1025*	Glycine betaine ABC transport system, permease protein	*gbuB*	+	*lmo1015*	19	0.04	**0.48**	**0.75**	**1.14**	0.08
*LMON_1026*	Glycine betaine ABC transport system, glycine betaine-binding protein	*gbuC*	+	*lmo1016*	15	0.10	**0.54**	**0.64**	0.87	**0.71**
*LMON_1488*	Osmotically activated L-carnitine/choline ABC transporter, permease protein OpuCD	*opuCD*	−	*lmo1425*	10	**2.34**	**5.37**	**3.52**	**1.79**	**1.29**
*LMON_1489*	Osmotically activated L-carnitine/choline ABC transporter, substrate-binding protein OpuCC	*opuCC*	−	*lmo1426*	10	**2.45**	**5.23**	**3.78**	**1.82**	**1.24**
*LMON_1490*	Osmotically activated L-carnitine/choline ABC transporter, permease protein OpuCB	*opuCB*	−	*lmo1427*	10	**2.51**	**5.28**	**3.78**	**1.58**	**1.10**
*LMON_1491*	Osmotically activated L-carnitine/choline ABC transporter, ATP-binding protein OpuCA	*opuCA*	−	*lmo1428*	10	**2.63**	**5.54**	**3.94**	**1.30**	-0.01
*LMON_2268*	Oligopeptide transport ATP-binding protein OppF	*oppF*	−	*lmo2192*	2	**-0.56**	**-0.61**	**-0.43**	-0.39	**1.39**
*LMON_2269*	Oligopeptide transport ATP-binding protein OppD	*oppD*	−	*lmo2193*	2	**-0.59**	**-0.74**	**-0.38**	-0.30	**0.91**
*LMON_2270*	Oligopeptide transport system permease protein OppC	*oppC*	−	*lmo2194*	15*	**-0.66**	**-0.91**	**-0.35**	-0.15	-0.29
*LMON_2271*	Oligopeptide transport system permease protein OppB	*oppB*	−	*lmo2195*	19	**-0.61**	**-0.85**	**-0.35**	0.09	**-0.79**
*LMON_2272*	Oligopeptide ABC transporter, periplasmic oligopeptide-binding protein OppA	*oppA*	−	*lmo2196*	9	**-0.67**	0.00	**-0.46**	**-1.44**	-0.17
**RNA and DNA repair**										
*LMON_0004*	Hypothetical protein		+	*lmo0004*	16	**1.25**	**0.69**	**0.54**	-0.29	-0.69
*LMON_0005*	DNA recombination and repair protein RecF	*recF*	+	*lmo0005*	16	**1.40**	**0.74**	**0.59**	0.06	-0.15
*LMON_0006*	DNA gyrase subunit B	*gyrB*	+	*lmo0006*	20	**1.24**	**0.53**	**0.49**	-0.15	0.45
*LMON_0007*	DNA gyrase subunit A	*gyrA*	+	*lmo0007*	20	**1.17**	**0.61**	**0.40**	-0.10	**1.15**
*LMON_0846*	Endoribonuclease L-PSP		+	*lmo0844*	7/6/10	**0.53**	**1.18**	**1.11**	0.24	-0.41
*LMON_0848*	Excinuclease ABC subunit C		+	*lmo0846*	15	**-0.44**	-0.04	0.15	**1.76**	**1.12**
*LMON_0869*	Cold-shock DEAD-box protein A		+	*lmo0866*	14	**1.97**	**0.85**	0.38	**1.87**	0.45
*LMON_1037*	Ribonuclease J1 (endonuclease and 5' exonuclease)		−	*lmo1027*	16	**1.71**	**0.33**	-0.03	-0.10	**-1.17**
*LMON_1225*	DNA polymerase X family		+	*lmo1231*	2	**1.12**	**0.22**	**0.42**	**0.76**	**2.10**
*LMON_1305*	ATP-dependent RNA helicase YxiN		+	*lmo1246*	6	**0.99**	**0.75**	**0.56**	0.07	**-1.04**
*LMON_1317*	Hypothetical protein		+	*lmo1256*	12	**2.04**	**1.68**	**1.00**	**-1.20**	**2.14**
*LMON_1336*	DNA topoisomerase I	*topA*	+	*lmo1275*	4	0.02	**0.74**	**0.30**	**1.00**	0.31
*LMON_1348*	Topoisomerase IV subunit A	*parC*	+	*lmo1287*	2	-0.02	-0.06	0.19	0.10	**1.39**
*LMON_1461*	RecA protein	*recA*	+	*lmo1398*	12	**0.73**	**1.27**	**0.34**	-0.27	0.46
*LMON_1513*	ATP-dependent RNA helicase YqfR		−	*lmo1450*	6	**1.33**	**1.12**	**1.18**	**1.01**	**-1.04**
*LMON_1787*	ATP-dependent RNA helicase YfmL		−	*lmo1722*	6	**1.69**	**1.45**	**1.28**	**1.21**	**0.47**
*LMON_2342*	ATP-dependent nuclease, subunit A		−	*lmo2267*	17	**-0.99**	**-0.77**	**-0.35**	**-0.62**	**1.77**
*LMON_2343*	ATP-dependent nuclease, subunit B	*addB*	−	*lmo2268*	17	**-0.78**	**-0.45**	-0.11	-0.60	**1.42**
*LMON_2778*	DNA topoisomerase III	*topB*	−	*lmo2756*	17	-0.25	-0.03	**0.19**	-0.20	**1.40**
*LMON_2812*	DNA-binding protein			*lmo2792*	5	**0.62**	**1.34**	**0.75**	**-0.80**	**2.49**
**Regulatory elements**										
*LMON_0198*	Virulence regulatory factor PrfA	*prfA*	−	*lmo0200*	17	**-2.04**	**-0.44**	**1.01**	0.10	**5.32**
*LMON_0229*	Transcriptional regulator CtsR	*ctsR*	+	*lmo0229*	16	**1.90**	**0.42**	**0.47**	-0.55	0.16
*LMON_0244*	RNA polymerase sigma factor SigH	*sigH*	+	*lmo0243*	7	**-1.29**	**-0.60**	**-0.49**	-0.59	**-2.35**
*LMON_0431*	RNA polymerase sigma factor SigC	*sigC*	−	*lmo0423*	13	**-2.03**	**-1.48**	-0.59	-0.62	**-0.97**
*LMON_0679*	Motility gene repressor MogR	*mogR*	−	*lmo0674*	16	**0.62**	0.02	0.03	**-1.20**	**-0.72**
*LMON_0900*	RNA polymerase sigma factor SigB	*sigB*	+	*lmo0895*	10	0.00	**0.58**	0.19	**-0.78**	**-0.62**
*LMON_1166*	Ethanolamine sensory transduction histidine kinase		+	*lmo1173*	5/2	**1.03**	**0.71**	-0.03	0.06	**2.43**
*LMON_1341*	GTP-sensing transcriptional pleiotropic repressor codY	*codY*	+	*lmo1280*	3	**-0.42**	**0.61**	0.02	0.09	**1.27**
*LMON_1517*	RNA polymerase sigma factor RpoD	*rpoD*	−	*lmo1454*	5	0.08	**-0.24**	**-0.60**	**-0.88**	0.54
*LMON_1539*	Heat-inducible transcription repressor HrcA	*hrcA*	−	*lmo1475*	16	**1.42**	-0.02	0.10	-0.28	**-0.43**
*LMON_1807*	Two-component sensor histidine kinase BceS	*cesK*	−	*lmo1741*	2	-0.17	-0.17	0.02	0.03	**2.05**
*LMON_1811*	Two-component response regulator YvcP (VirR in L. monocytogenes 10403S)	*virR*	−	*lmo1745*	17	**-0.48**	**-0.61**	-0.12	-0.30	0.02
*LMON_2432*	Sensor histidine kinase	*virS*	−	*lmo2421*	14	**1.27**	-0.03	0.28	**0.89**	0.17
*LMON_2472*	RNA polymerase sigma factor SigL	*sigL*	−	*lmo2461*	5	-0.20	-0.05	-0.10	-0.42	**0.85**
*LMON_2527*	Transcriptional regulator DegU	*degU*	−	*lmo2515*	16	**1.61**	**0.62**	**0.54**	0.04	**-0.94**
*LMON_2702*	Osmosensitive K+ channel histidine kinase KdpD	*kdpD*	−	*lmo2679*	2	**0.99**	**0.46**	**0.60**	0.44	**4.22**
**Ribosome functions**										
*LMON_0209*	LSU ribosomal protein L25p	*ctc*	+	*lmo0211*	10	**2.16**	**3.43**	**2.72**	**0.87**	**1.58**
*LMON_0486*	LSU ribosomal protein L32p	*rpmF*	+	*lmo0486*	19	**1.43**	**1.46**	**1.76**	**1.75**	**1.22**
*LMON_1388*	Translation initiation factor 2	*infB*	+	*lmo1325*	2	**0.55**	0.09	-0.01	0.39	**1.16**
*LMON_1390*	Ribosome-binding factor A	*rbfA*	+	*lmo1327*	2	**0.59**	0.16	-0.03	0.25	**1.75**
*LMON_2523*	Ribosomal subunit interface protein		−	*lmo2511*	5	**1.08**	**1.32**	**0.65**	**-0.90**	**3.14**
**Cold-stress proteins**										
*LMON_1427*	Cold-shock protein	*cspL*	+	*lmo1364*	16	**1.58**	**1.34**	**0.45**	0.12	0.27
*LMON_1947*	Cold shock protein CspD	*cspD*	+	*lmo1879*	12	**0.96**	**1.00**	-0.09	**-3.93**	**-1.96**
*LMON_2087*	Cold shock protein CspB	*cspB*	−	*lmo2016*	16	**-3.67**	**-4.42**	**-5.85**	**-7.83**	**-2.97**
**Additional proteins**										
*LMON_0213*	Low temperature requirement B protein	*ltrB*	+	*lmo0215*	2	0.03	**-0.26**	**-0.25**	0.00	**1.78**
*LMON_0398*	Low temperature requirement protein A	*ltrA*	−	*lmo0389*	12*	**0.43**	-0.23	**0.61**	-0.63	0.08
*LMON_0691*	Flagellar motor rotation protein MotB	*motB*	+	*lmo0686*	17	**-0.81**	**-0.37**	0.07	-0.26	**1.44**
*LMON_0693*	Glycosyl transferase	*gmaR*	+	*lmo0688*	17	**-0.84**	**-0.42**	-0.06	-0.44	**1.29**
*LMON_0694*	Chemotaxis protein CheV	*cheV*	+	*lmo0689*	17	-0.77	-0.04	**-0.19**	**-0.65**	**2.37**
*LMON_0695*	Flagellin protein FlaA	*flaA*	+	*lmo0690*	5	0.58	**-1.03**	**-0.70**	**-2.00**	**1.16**
*LMON_0950*	Non-specific DNA-binding protein Dps / Iron-binding ferritin-like antioxidant protein / Ferroxidase	*fri*	+	*lmo0943*	10	0.39	**1.50**	0.03	-0.51	-0.22
*LMON_2409*	Low temperature requirement C protein	*ltrC*	+	*lmo2398*	12	**1.14**	**1.21**	**0.75**	**-1.61**	0.16

* indicates <0.50 cluster membership; G1–G5 refer to differential expression in *L*. *monocytogenes* cells grown at 4°C relative to 20°C and across five specific growth phases (see Fig 1); Blue shading indicates genes with significantly increased (> 1log_2_, p<0.05) gene expression and yellow shading indicates genes with significantly decreased (< -1log_2_, p<0.05) expression at 4°C relative to 20°C; Bolded values indicate significant (p<0.05) differential expression changes.

Oligopeptide uptake in *L*. *monocytogenes* is mediated by the membrane permease OppA (*oppABCDF*) which transports peptides containing up to eight residues [23]. This transporter appears to contribute to *L*. *monocytogenes* cold tolerance as an *oppA* null mutant displayed reduced growth at 5°C in BHI medium [23]. Previously, Durack et al. [12] observed upregulation of *oppBCF* in late exponential early stationary-phase cells at 4°C while Chan et al. [11] found that only *oppA* levels were elevated in stationary-phase cells. Contrary to these reports, we did not observe any notable DE of the *opp* operon, except for a 2.6-fold induction of *oppF* at G5 (Table 6). While the operon may be necessary for low temperature growth, it does not appear to have increased transcription at 4°C relative to 20°C in our cold tolerant strain.

#### RNA and DNA repair

At low temperatures, both DNA replication and transcription are hindered by cold-induced changes in nucleic acid structures. L. monocytogenes appears to respond to these challenges by upregulating genes encoding topoisomerases and DNA gyrases, which help maintain the superhelical tension of DNA; RNA helicases, which unwind secondary RNA structures; and exo- and endonucleases, which function in nucleic acid repair [11, 12, 15]. Markkula et al. [15] reported the upregulation of four DEAD-box RNA helicase genes (lmo0866, lmo1246, lmo1450, lmo1722) in L. monocytogenes for up to 7 h following a downshift from 37°C to 5°C. They also found that, compared with the wildtype strain, null mutants of lmo0866, lmo1450, and lmo1722 displayed restricted growth at 3°C. Similarly, we observed upregulation of lmo1450 (LMON_1513) and lmo1722 (LMON_1787) at G1–G4, and lmo0866 (LMON_0869) at G1:G4 (Table 6). Also in agreement with Markkula et al. [15], lmo1246 (LMON_1305) appeared to be the least important of the four genes, with <2-fold induced expression at G1–G3.

Several DNA gyrase and topoisomerase genes were also upregulated in our study. Notably, *topA* was upregulated at G4, and *topB* and *parC* were upregulated at G5 (Table 6). In *L*. *monocytogenes*, the DNA gyrase genes *gyrB* and *gyrA* are in a four-gene operon, along with a gene encoding a hypothetical protein, and *recF*, a DNA recombination and repair protein. All four genes were upregulated at G1, and *gyrA* was also induced at G5. Upregulation of another DNA recombination and repair protein, *recA*, was also observed at G2.

Among the nucleic acid repair-proteins induced in this study were ribonuclease J1 (*LMON*_*1037*) at G1, an endoribonuclease (*LMON*_*0846*) at G2–G3, two nuclease subunits (*LMON*_*2342–2343*) at G5, and an excinuclease (*LMON*_*0848*) at G4–G5 (Table 6). Additionally, an X family DNA polymerase (*LMON*_*1225*) was induced at G1:G5. These polymerases synthesize unusual DNA structures that may serve as indicators of the induction of DNA repair [81–83]. A DNA-binding protein (*LMON*_*2812*) with a putative role in oxidative-damage protection [84] was also upregulated at G2 and G5. Lastly, a hypothetical protein (*LMON*_*1317*) containing a nudix hydrolase domain was upregulated at G1–G3:G5 (Table 6). Some members of this protein family are known to degrade oxidatively damaged nucleoside di- and triphosphates, while other members control the levels of metabolic intermediates and signaling compounds [85].

Overall, genes associated with RNA and DNA repair were predominantly cold-induced in lag- and stationary-phase cells (G1 and G5). This is expected given that cold-induced damage to nucleic acids is highest directly following cold stress, and nutrient-depleted stationary-phase cultures experience higher mutation rates and increased levels of oxidative damage [86–89].

#### Regulatory elements

Many mutant characterization and transcriptome studies have evaluated the roles of alternative sigma factors (σ^B^, σ^C^, σ^H^, σ^L^), two-component regulatory systems (TCRSs), and negative regulators in the *L*. *monocytogenes* CSR. Of the four alternative sigma factors, σ^B^ has been the most extensively studied. It positively regulates over 100 genes when the organism enters stationary phase or is subjected to environmental stresses including low pH, high salt, or carbon starvation [90–94]. Although induced expression of *sigB* has been observed in *L*. *monocytogenes* up to 12 h following cold stress [11, 17, 19], the σ^B^ regulon is predominantly downregulated during cold stress [11, 12], demonstrating that increased *sigB* expression does not necessarily correlate with increased σ^B^ activity. Furthermore, compared with wildtype strains, *sigB* null mutants show little to no difference in cold tolerance [16, 19, 20, 95], indicating that *sigB* is not critical for cold-stress survival. In the present study, the DE pattern of *sigB* followed that of cluster 10 (Fig 5), with a maximum 1.5-fold increase in expression at G2 (Table 6). Similar expression patterns were observed for three additional *sigB* operon genes (rsbWVX) while the first four genes (rsbRSTU) of the eight-gene operon were not induced under cold stress. Despite low levels of *sigB* induction, we found that σ^B^-dependent genes were overrepresented (p<0.05) among the cold-induced genes at all growth phases (Table 7). Examples of such genes include *fri*, *bsh*, *dapE*, *uspA*, *gabD*, *opuCABCD*, *mpoBACD*, *phoU*, *csbD*, *ltrC*, and *hrcA*. Although some of these genes are solely activated by σ^B^, others are co-regulated by other unknown or known transcription factors such as σ^L^, σ^H^, and PrfA [16, 20, 96–98]. As previously mentioned, σ^B^ is also activated upon entry into stationary phase [99–101]. Consistent with this fact, we observed that *sigB* transcript levels during growth at 20°C increased from 4.5k mapped reads at C1 to 15k at C5 (S2 Table).

**Table 7:** Transcription regulators significantly (p<0.05*) overrepresented among genes differentially expressed at 4°C vs. 20°C.

Regulators	Regulon gene examples	p-value (# of contributing genes)				
		G1	G2	G3	G4	G5
**Regulons upregulated**						
σ^B^	*inlA*, *bsh*, *mpoABCD*, *gabDE*, *opuCABCD*, *ltrC*, *csbD*, *phoU*, *hrcA*	3.70E-10 (63)	3.95E-23 (69)		1.26E-2 (8)	9.58E-4 (51)
CodY	*argBDFJ*			1.96E-2 (4)		4.70E-2 (4)
CtsR	*clpBCEP*, *mcsAB*, *ctsR*, *gpmA*	1.74E-4 (8)				
HrcA	*hrcA*, *grpE*, *dnaK*	4.14E-2 (3)				
**Regulons downregulated**						
RpoD	*dltABCD*, *plcAB*, *hly*, *mpl*, *actA*, *pmk*, *pgdA*, *ctaP*, *arcB*	1.91E-2 (24)	4.57E-2 (16)	1.05E-2 (14)		1.59E-2 (39)
VirR	*dltABCD*	2.15E-2 (4)	3.50E-4 (5)	1.66E-3 (4)		
PrfA	*prfA*, *mpl*, *actA*, *plcB*, *inlC*, *hly*	1.16E-2 (5)		4.44E-4 (5)		3.14E-2 (6)
σ^B^	*mogR*, *mpoAB*, *phoU*, *ltrC*, *arsC*, *uspA*				2.14E-3 (52)	
MogR	*fliNPQRK*, *cheRY*, *flhB*, *flgDE*					1.35E-2 (11)

* Statistical overrepresentation of gene sets were determined using Fisher’s exact test and the BioCyc database.

The alternative sigma factor σ^L^ has been shown to contribute to the ability of *L*. *monocytogenes* to tolerate cold [20, 102], osmotic, and acid stress [102, 103]. This sigma factor positively regulates >400 genes, including those involved in cell envelope synthesis, motility, and PTS sugar uptake and catabolism [28, 104]. Although *sigL* induction has been reported in exponential and late exponential early stationary-phase cells of cold-adapted *L*. *monocytogenes* [12, 27, 102], deleting *sigL* does not impact the ability of *L*. *monocytogenes* ability to grow at cold temperatures [20, 28]. In the present study, the DE pattern of *sigL* followed that of cluster 5 (Fig 5), with baseline expression at G1–G4 and <2-fold increased expression at G5 (Table 6). Sixteen genes previously identified as being positively regulated by σ^L^ in *L*. *monocytogenes* at 3°C [28] also exhibited similar DE patterns.

The remaining alternative sigma factors, σ^H^ and σ^C^, contribute to the survival of *L*. *monocytogenes* under alkaline and heat stress, respectively [105–107]. However, these sigma factors have limited roles in cold tolerance [20]. Consistent with previous findings, we did not observe the upregulation of *sigH* or *sigC* in our study.

RpoD is the principal RNA polymerase sigma factor in *L*. *monocytogenes* and regulates housekeeping genes associated with ribosome structure, protein synthesis, and rRNA and tRNA [108]. Like the DE pattern for *sigL*, the DE pattern of *rpoD* followed that of cluster 5 (Fig 5), with a maximum 1.5-fold increase in expression at G5 (Table 6). Accordingly, genes positively regulated by RpoD were overrepresented (p<0.05) among the downregulated genes at all growth phases except G4. Many genes in the RpoD regulon are co-activated by PrfA, and correspondingly, PrfA-regulated genes were also overrepresented among the downregulated genes at G1–G3:G5 (Table 7). Genes co-regulated by RpoD and PrfA include *prfA*, *plcAB*, *hly*, *mpl*, *inlC*, and *actA*, all of which are associated with virulence [109]. Other studies have also reported lower levels of PrfA-regulated virulence genes in cold-adapted exponential and stationary-phase cells [11, 12, 110]. In the present study, the DE pattern of *prfA* fit that of cluster 17 (Fig 5), with 2-fold and 40-fold increased expression at G3 and G5, respectively (Table 6). Interestingly, although the transcription of *prfA* increased dramatically at G5, the expression levels of many PrfA-dependent virulence genes remained strongly downregulated. Researchers have proposed that PrfA activity, but not *prfA* transcription, is inhibited by unphosphorylated forms of PTS permeases that occur during active sugar transport; these PTS permeases may bind directly to PrfA [111–113]. Our results support this hypothesis, as many PTS operons and other carbohydrate uptake and catabolism genes were upregulated at G5 and belonged to the same cluster profile as *prfA* (Fig 5, cluster 17). Whether PrfA is subsequently involved in regulating these genes is still unclear.

CodY is a pleiotropic transcriptional regulator that actively represses the transcription of genes involved in amino acid metabolism, nitrogen assimilation, mobility and chemotaxis, and sugar uptake, among others [114]. In agreement with previous work [11], we observed the highest induction (2.4-fold) of *codY* in at G5 following the DE pattern shown in cluster 3 (Fig 5).

DegU is another well-known response regulator that regulates the expression of motility-, virulence-, and biofilm-related genes in *L*. *monocytogenes* [29, 72, 115]. Our results showed that *degU* induction was greatest (3-fold) at G1 (Table 6), with a DE pattern consistent with that of cluster 16 (Fig 5). This probably explains why increased expression of *degU* was not observed in previous cold stress transcriptome studies of *L*. *monocytogenes*, which focused on exponential- and stationary-phase cells [11, 12]. Despite the induction of *degU* at G1, the flagella operon (*LMON*_*0680–0694*) that the DegU protein regulates remained suppressed, likely due to co-induction of the gene encoding the transcriptional repressor MogR, which shared the same DE pattern as *degU*. In *B*. *subtilis*, DegU is part of the two-component system DegS/U, which regulates the expression of genes encoding various extracellular enzymes [116]. This suggests that DegU may also contribute to the regulation of extracellular enzymes in *L*. *monocytogenes* that facilitate cold growth.

The negative regulators HrcA and CtsR repress expression of several genes for heat-shock proteins and cellular protein quality control (e.g. *dnaK*, *grpE*, *groES*, *groEL*, *clpB*, *clpC*, *clpE*, *clpP*) in *L*. *monocytogenes* and other bacteria [117–119]. Increased expression of many of these genes has also been reported in response to salt, cold, and ethanol stress [27, 120, 121]. In the present study, we found that *hrcA* and *ctsR*, like *degU* and *mogR*, were both maximally upregulated at G1, as were many of the genes they regulate. This was somewhat surprising given the inverse relationship expected between the DE patterns of transcription repressors and their gene targets. However, CtsR and HrcA regulons are known to have a considerable amount of overlap with PrfA and σ^B^ regulons, demonstrating the complexity and fine-tuning abilities of bacterial regulatory networks, which allow bacteria to survive a wide-range of conditions [98, 122, 123].

Two-component signaling systems (TCSs) are also important regulators of bacterial stress responses and typically consist of a transmembrane sensor histidine kinase (HK), and a cognate cytoplasmic response regulator [124–127]. The sequenced genome of *L*. *monocytogenes* EGD-e contains 16 known TCSs [128]. Using mutant characterization, Pöntinen et al. [129] showed that only the HKs LisK and YycG of the LisKR and YycGF TCSs, respectively, are important for *L*. *monocytogenes* growth under cold stress. However, increased expression of *lisK* was not observed in either the Pöntinen et al. [129] study or the present study, highlighting that the importance of a gene does not necessarily correlate with its level of induction. HKs with increased expression in our study included *cesK* and *LMON*_*1166* at G1, and *kdpD*, *LMON*_*1166*, and *virS* at G5 (Table 6). Of these, *kdpD*, which encodes the HK for an osmosensitive K+ channel, exhibited the largest increase in expression (18.6-fold). KdpD, together with its response regulator KdpE, controls the expression of a high-affinity K+ translocating ATPase [130, 131]. Among the many K+ transport systems in bacteria, Kdp has the highest affinity for K+ and it is only expressed when other systems are unable to meet the cell’s K+ needs. Our results therefore suggest that activation of Kdp is an important mechanism used by *L*. *monocytogenes* to survive long-term exposure to cold stress.

#### Ribosome functions

When bacteria are subjected to a temperature downshift, their ribosome structures become compromised, resulting in translation inhibition and a prolonged lag phase. In fact, inhibition of ribosomal functions induces CSR proteins [132]. In E. coli, three ribosome-associated proteins (IF2, CsdA, RbfA) are required for protein synthesis and subsequent cell growth at low temperatures [133–135]. In contrast, 22 other ribosomal proteins are not believed to be essential for the growth of *E*. *coli* or *B*. *subtilis* at low temperatures [136, 137]. In *L*. *monocytogenes*, the role of ribosomes and their associated proteins in cold stress remains largely unknown. In a study by Durack et al. [12], ribosome protein genes were strongly activated in osmo- and cold-adapted *L*. *monocytogenes* cells. In our study, of the 58 ribosomal proteins identified in *L*. *monocytogenes* EGD, 22 were upregulated exclusively at G5 in the cold tolerant- strain we studied. Among these genes were those encoding *L*. *monocytogenes* homologs of IF2 (*infB*) and RbfA, previously mentioned for their roles in stabilizing cold-sensitive ribosomes in *E*. *coli*. An additional two ribosomal protein genes, *ctc* and *rpmF*, were significantly upregulated at all growth phases, and a ribosomal subunit interface protein (LMON_2523) was induced at G1–G2:G5 (Table 6). In *B*. *subtilis*, ctc is induced in response to osmotic, heat, and oxidative stress [138, 139], and ithas similarly been linked to osmo- and cold-tolerance in *L*. *monocytogenes* [12, 56].

In bacteria, rRNA and ribosomal protein synthesis are tightly controlled, to meet the translational needs of the cell. The increased transcription of ribosome proteins in cold-adapted stationary-phase cells may reflect an increased demand for protein synthesis, as suggested by the large number of strongly upregulated genes at G5. Alternatively, ribosomal proteins have been shown to participate in extra-ribosomal functions, as independent polypeptides with roles in transcription and DNA repair [140–142]. Thus, they might contribute to the *L*. *monocytogenes* CSR in yet undetermined ways.

#### Cold-stress proteins

CSPs are a conserved family of small (~70 aa) proteins containing a nucleic acid-binding domain. Found in many prokaryotic and eukaryotic organisms, CSPs can act as transcriptional activators, antiterminators, or as RNA chaperones that enhance translation at low temperatures by blocking the development of secondary mRNA structures [10, 70, 71]. Three CSPs have been identified in *L*. *monocytogenes* and are listed here in the order of functional importance: CspL>CspD>CspB [143]. Furthermore, *L*. *monocytogenes* CSPs appear to be only induced during the early stages following cold stress [11, 12, 143]. In the present study, *cspB* was downregulated at all growth phases, reaching a maximum 228-fold decrease at G5, while *cspD* and *cspL* were upregulated at G1–G2 and then exhibited no change or decreased expression at the later growth phases (Table 6).

#### Additional proteins with putative roles in the *L*. *monocytogenes* cold-stress response

A few other genes have also been associated with the *L*. *monocytogenes* CSR. Zheng and Kathariou [144, 145] identified three low temperature requirement proteins (LtrA, LtrB, and LtrC) necessary for growth at cold temperatures. However, varying results have been reported regarding the expression of these genes at low temperatures. Pieta et al. [14] observed higher levels of *ltrC* transcripts at 7°C than at 37°C in exponential-phase cells, whereas other researchers saw either no difference in expression or decreased expression in exponential- and stationary-phase cells of cold-adapted *L*. *monocytogenes* [11, 12, 27]. Increased expression of *ltrC* has also been reported in *L*. *monocytogenes* EGD-e, directly following an upshift in temperature from 37°C to 48°C [146]. Importantly, we observed increased expression of *ltrC* only in the early stages following cold stress (G1 and G2), which may partly explain why no differences were seen in studies that analyzed exponential- and stationary-phase cells. As for other low temperature requirement protein genes, *ltrB* was upregulated at G5 while no notable changes were seen for *ltrA*.

The *fri* (*flp*) gene, which encodes ferritin, is also commonly discussed in cold stress studies of *L*. *monocytogenes* and is hypothesized to play a role in iron storage [96, 146, 147]. Previous studies have reported that *fri* is induced upon entry into stationary phase in *L*. *monocytogenes* subjected to low temperatures [27, 50, 96]. Additionally, *fri* null mutants display reduced growth at 4°C in BHIB and increased sensitivity to oxidative stress [146, 148]. In our study, *fri* was exclusively induced at G2 (Table 6). However, in agreement with the findings of Polidoro et al. [96], the number of *fri* transcripts at 20°C was nine times higher in stationary-phase cells than in lag-phase cells.

Liu et al. [22] identified a membrane-associated phosphohydrolase *(pgpH)* as the interrupted gene in an *L*. *monocytogenes* cold-sensitive transposon mutant. Compared with the parent strain, this mutant also showed increased intracellular levels of the phosphorylated guanosine nucleotide (p)ppGpp, suggesting that PgpH may be critical for (p)ppGpp degradation at low temperatures. Cellular (p)ppGpp inhibits RNA synthesis when a shortage of amino acids is present. Such conditions can occur during cold stress, because of structural damage to membrane transporters and intracellular enzymes. Arguedas-Villa et al. [17] reported increased *pgpH* expression in cold-tolerant but not cold-sensitive strains of *L*. *monocytogenes* at 4°C compared to 37°C. Though we also evaluated the gene expression of a cold-tolerant strain, we observed no significant induction of *pgpH* (*LMON*_*1529*) at 4°C relative to 20°C.

The induction of flagella biosynthesis and motility-related genes has frequently been reported in *L*. *monocytogenes* following a temperature downshift [11, 14, 27]. However, the reliability of these observations is debatable as 37°C was uniformly used as the control temperature and *L*. *monocytogenes* is not typically motile above 30°C [149, 150]. Recently, Cordero et al. [13] reported that fast cold-growing *L*. *monocytogenes* strains are less motile than slow cold-growing strains. They hypothesized that low motility might allow cold-tolerant strains to proliferate more rapidly at low temperature. In the present study, our cold-tolerant strain exhibited decreased expression of most flagella operons at all growth phases. However, five motility-specific genes (*LMON*_*0691–0695*) were upregulated at G5, including *motB*, *gmaR*, *cheV*, and *flaA* (Table 6). The role of motility genes in prolonged cold-stress survival remain speculative, but one hypothesis is that it is beneficial for cells to be able to move to environments more suitable in terms of nutrition or other requirements.

### Cold-induced membrane lipid composition changes

To gain a better understanding of both the timing associated with cold-induced membrane FA changes in *L*. *monocytogenes* and the types of changes that occur, we analyzed FAs extracted from cells at the same time points used in our RNA-seq experiment.

#### Increase in anteiso C15:0

The *L*. *monocytogenes* lipid membrane consists predominantly of BCFAs (~90% of total FAs). The four most abundant BCFAs, listed in decreasing order, are anteiso-C15:0 (a-C15:0), a-C17:0, iso-C15:0 and i-C17:0 [24, 51, 151, 152]. When *L*. *monocytogenes* experiences a decrease in temperature, it increases the relative proportion of a-C15:0 at the expense of a-C17:0 [24, 52]. Depending on the length of the BCFA and the bacterial strain studied, a switch from iso to anteiso FAs can also occur (ie. i-C17:0 to a-C17:0).

To date, the membrane FA profile of *L*. *monocytogenes* under cold stresses has primarily been investigated using late exponential to early stationary-phase cells [24, 51, 151, 152]. In most cases, researchers have observed a ~20% increase in the relative proportion of a-C15:0 among cold-grown cells (5–10°C), with maximum levels ranging from 66–80%. Correspondingly, a-C17:0 levels decrease by 20–25% with minimum levels ranging from 3–14%.

**Fig 6:**
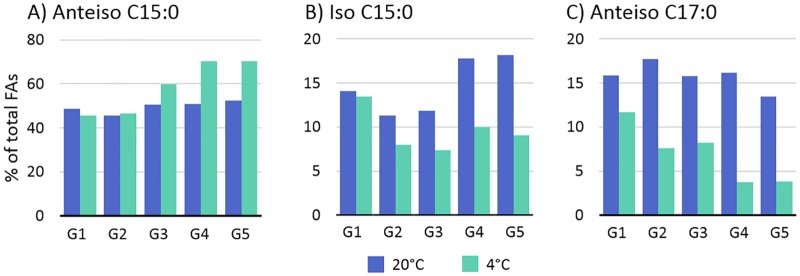
Relative proportions of specific branched-chain fatty acids (FAs) out of total FAs found in *L*. *monocytogenes* cells harvested across five growth phases at 4°C and 20°C. A) anteiso C15:0, B) iso C15:0, and C) anteiso C17:0. Lipids were extracted from cell concentrates and resulting fatty acid methyl esters (FAME) were analyzed by gas chromatography. See Fig 1 for information about growth phases.

In the present study, a-C15:0 levels increased from 46% at T1 to a maximum of 70% at T4 (Fig 6A), whereas levels remained constant around 50% in 20°C-grown cells. Thus, in this study, the first one to look at cold-induced membrane FA changes at multiple growth phases, we show that *L*. *monocytogenes* continues to make alterations until it transitions into stationary phase (T4), at which point minimal further adjustments are made. Correspondingly, a-C17:0 levels decreased from 12% at T1 to 4% at T4, whereas at 20°C they ranged from 13–18% (Fig 6C). Levels of i-C16:0 and iC15:0 also decreased by 2.3% and 9%, respectively at 4°C (Fig 6B). Additional changes in the BCFA composition of cold-stressed cells included a 2.5–2.9% increase in the proportion of i-C14:0 and the appearance of i-C13:0 and a-C13:0.

As previously discussed, anteiso BCFAs are synthesized from the BCAAs isoleucine, and iso BCFAs are synthesized from leucine and valine [52]. These BCAAs are produced by proteins encoded by a nine-gene operon (*LMON*_*2054–2062*). At 4°C, we found that all genes in this operon were induced >4-fold at all growth phases. Interestingly, genes from the BKD operon (*LMON*_*1432–1437*) which encodes enzymes that convert BCAAs into BCFAs, showed either no change in expression or decreased expression at 4°C. Other researchers have also noted a lack of *bkd* induction in *L*. *monocytogenes* under cold-stress conditions [24, 51]. A BKD-independent pathway may exist, in which BCFA synthesis uses branched-chain α-keto acids instead of branched-chain acyl-CoA esters as primers [52]. Alternatively, Nickel and colleagues [153] showed that in *B*. *subtilis* the promoter regions of five operons, including BKD, are not cold-inducible but exhibited increased mRNA stability at low temperatures.

In addition to the BKD operon, many other FA synthesis genes were also downregulated at 4°C (e.g. *fabD*, *fabG*, *fabZ*, *acpP*, *plsX*, *plsY*). However, a *fabG* isozyme (*LMON*_*0351*) encoding a 3-oxoacyl-[acyl-carrier protein] reductase remained upregulated at G1–G3:G5. Other upregulated genes with known or putative functions in FA biosynthesis included two short-chain dehydrogenases (*LMON*_*0674* and *LMON*_*1898*), an oxidoreductase (*ylbE*), and a two-component response regulator (*LMON*_0289) with homology to a membrane FA regulator (YccF) in *Streptococcus pneumoniae* [154, 155].

#### Shortening of fatty acid chain lengths

**Fig 7:**
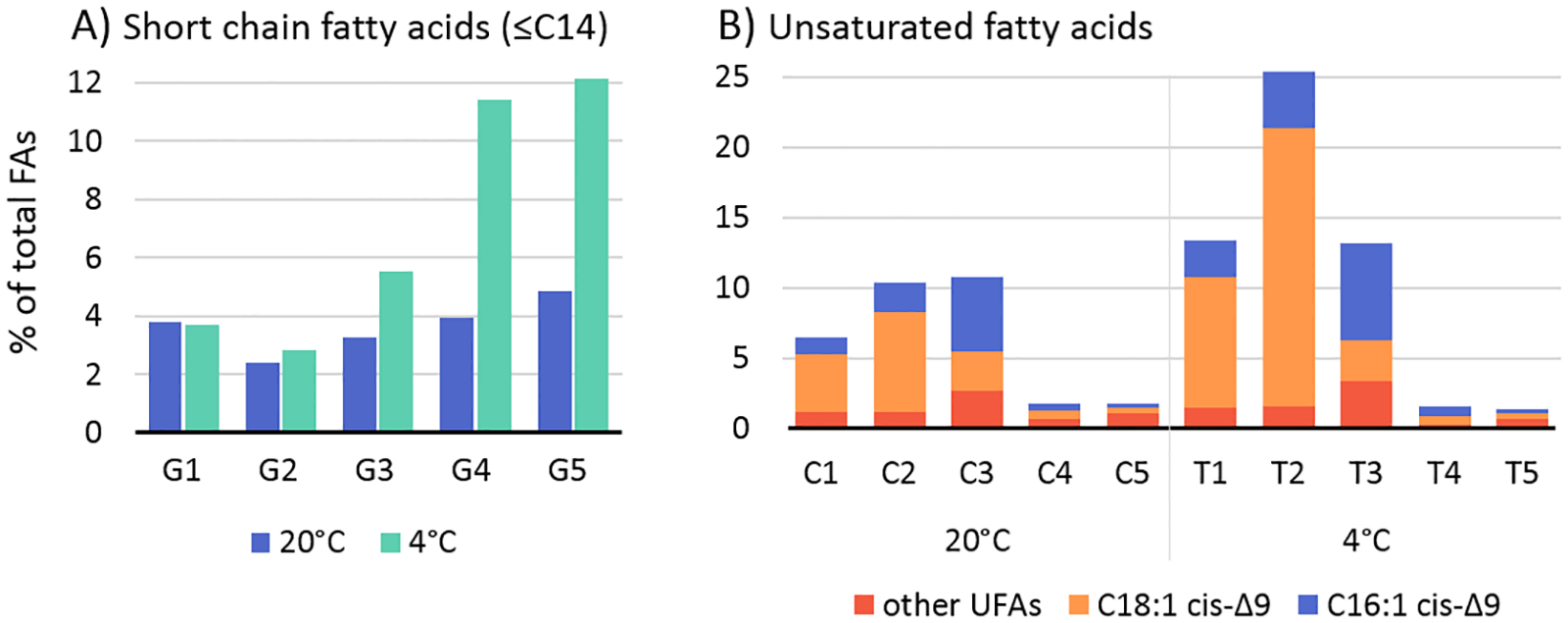
Relative proportion of short chain and unsaturated fatty acids (UFAs) out of total FAs found in *L*. *monocytogenes* cells harvested across five growth phases at 4 and 20°C. A) Proportion of short chain fatty acids containing ≤14 carbons, B) Proportion of C16:1 cis-Δ^9^ (palmitoleic acid), C18:1 cis-Δ^9^ (oleic acid), and all remaining UFAs. Lipids were extracted from cell concentrates and resulting fatty acid methyl esters (FAME) were analyzed by gas chromatography. See Fig 1 for information about growth phases.

Following a temperature downshift, L. monocytogenes incorporates FAs with shorter chain lengths to further decrease the phase-transition temperature of its membrane [156]. Overall, we observed an 8% increase in ≤C14 FAs at 4°C relative to 20°C, which became noticeable at T3 (Fig 7A). Moreover, by T5, 92% of Lm1’s membrane FAs contained fewer than 15 carbons; in 20°C grown cells, this percentage was 75%. Our results suggest that L. monocytogenes begins degrading unfavorable membrane phospholipids during lag phase but cannot synthesize its preferred FAs until growth resumes. This poses the question of how the cell retains adequate membrane fluidity during the early stages of cold stress. The answer to this question appears to involve the desaturation of existing FAs.

#### Increase in unsaturated fatty acids

Several bacterial species including E. coli, some species belonging to the phylum cyanobacteria, and some species belonging to the genus Bacillus, increase their proportion of membrane unsaturated FAs (UFAs) directly following a temperature downshift [157–159]. This process occurs rapidly due to the presence of membrane-associated desaturases that introduce double bonds into preexisting saturated FAs (SFAs). For example, in *E*. *coli*, cis-vaccenate is produced 30 seconds following a shift from 42°C to 24°C [160].

To date, the roles of UFAs in the *L*. *monocytogenes* CSR have been largely overshadowed by interests in BCFA modulation. Although some papers have reported the absence of UFAs in the *L*. *monocytogenes* membrane [24, 161–163], others have detected their presence in small amounts under various conditions [51, 151, 152, 162, 164–168]. In a study of *L*. *innocua*, levels of C18:1 FAs increased to 6.2% following a downshift from 35°C to 10°C [169]. Similarly, another study found that the *Bacillus megaterium* membrane contained a maximum of 22% C16:1 cis-Δ^9^ (palmitoleic acid) at 10°C, whereas the maximum was 2% at 35°C [53]. In the present study, the proportions of C16:1 cis-Δ^9^ and C18:1 cis-Δ^9^ (oleic acid) peaked at T3 (7%) and T2 (20%), respectively (Fig 7B). By contrast, no difference was detected between the proportions of oleic and palmitoleic acid in T4 and C4 cells, or T5 and C5 cells. Very small percentages (<1.2%) of other UFAs were also detected in our samples, but no notable differences were observed between the temperatures treatments (S3 Table). The fact that membrane UFA proportions only increased in cold-stressed lag- and exponential-phase cells likely explains why studies that have investigated late exponential- and stationary-phase cells have failed to detect any changes.

**Fig 8:**
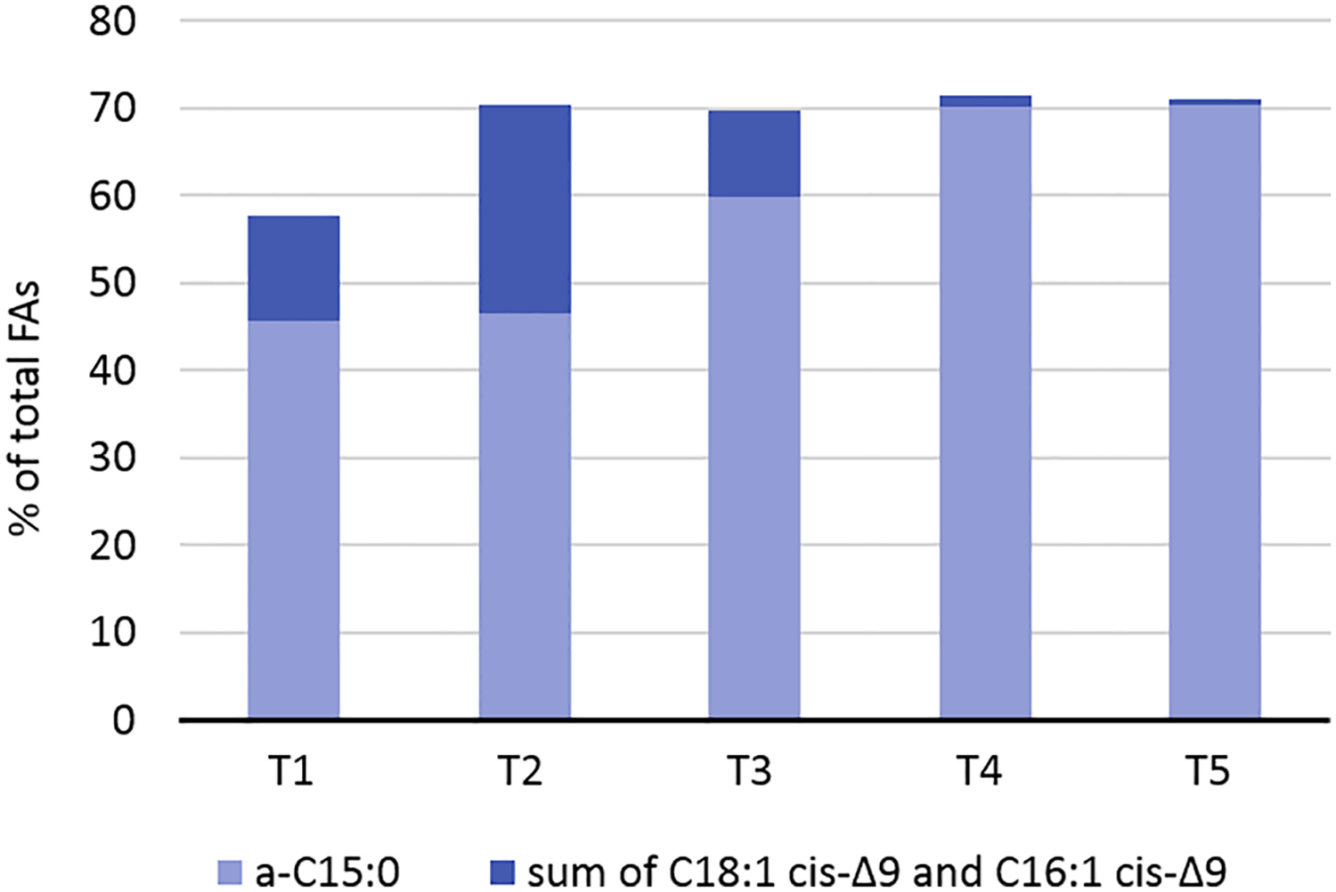
Proportions of anteiso C15:0 and the combined sum of the unsaturated fatty acids 16:1 cis-Δ^9^ and 18:1 cis-Δ^9^ out of total FAs found in *L*. *monocytogenes* cells harvested across five growth phases at 4°C. Lipids were extracted from cell concentrates and resulting fatty acid methyl esters (FAME) were analyzed by gas chromatography. See Fig 1 for information about growth phases.

Unlike the rapid conversion of SFAs to UFAs, switching from iso to anteiso BCFAs and from longer to shorter acyl chain FAs requires de novo synthesis of whole lipid molecules by cytoplasmic enzymes that are usually linked to growth [170]. Sato and Murata [171] showed that following a 10–15°C downshift in temperature, cyanobacterial cells could only resume growth and FA biosynthesis once a certain degree of membrane unsaturation was reached. Based on our findings, we suggest that in response to cold stress, *L*. *monocytogenes* converts C18:0 and C16:0 to oleic and palmitoleic acid, respectively, to rapidly lower its membrane phase-transition temperature. Fig 8 shows how UFA levels appear to compensate for a-C15:0 until its optimal levels are reached at T4. The centre placement of the cis-double bond in oleic and palmitoleic acid makes these FAs highly efficient at increasing membrane fluidity. For example, the average melting points of oleic and palmitoleic acid are 13°C and 1.22°C, respectively, compared to 24°C and 52°C for a-C15:0 and i-C15:0, respectively [172]. While oleic and palmitoleic acids are effective at rapidly increasing the membrane fluidity of *L*. *monocytogenes*, this membrane profile is probably less stable than one that includes a high proportion of a-C15:0. In support of this idea, Juneja and Davidson [173] showed that when provided with an external source of oleic acid, *L*. *monocytogenes* was able to increase the relative proportion of this acid in its membrane from 0.72 to 28%. However, these cells were highly susceptible to salt and several antimicrobials.

In addition to increasing in response to cold, UFA levels also increased up to 11% in lag and exponential-phase cells grown at 20°C (Fig 7B), suggesting a role for UFAs in regular cell growth. In *Saccharomyces cerevisiae*, oleic acid and ergosterol supplementation mitigate oxidative stress [174]. Similarly, bacteria transferred from a stationary-phase environment into a fresh oxygenated medium also experience oxidative stress [175] and may benefit from increased levels of membrane UFAs.

In *Bacillus*, phospholipid desaturases, encoded by *des*, are induced upon cold stress and controlled by a two-component regulator that senses changes in membrane fluidity [176, 177]. These desaturases introduce double bonds at the Δ^5^ or Δ^10^ positions of acyl chains attached to existing phospholipids. *Pseudomonas* has a similar desaturase system that can introduce double bonds at the Δ^9^ position [178]. Currently, no lipid desaturases have been identified in *Listeria*. However, Vadyvaloo et al. [166] showed that when *L*. *monocytogenes* is treated with a desaturase inhibitor, it exhibits decreased levels of membrane UFAs, demonstrating that such a system does exist. The synthesis of oleic (18:1 cis-Δ^9^) and palmitoletic (16:1 cis-Δ^9^) acids by *L*. *monocytogenes* suggests that it contains a desaturate system like that present in *Pseudomonas*. In the present study, we demonstrate the presence of additional UFAs, which contain double bonds in several other locations (S3 Table) highlighting the possibility that more than one desaturate system exists in the bacterium.

### Comparison of antisense transcription at 20°C and 4°C

A total of 92–261k paired-end (PE) reads were successfully mapped to the antisense strand of *L*. *monocytogenes* EGD ORFs, accounting for 0.61–1.63% of the total number of mapped reads. Overall, 70% of genes had >10 mapped PE asRNA reads, however, only 56 genes had >1,000 asRNA mapped PE reads with a maximum of 40,000 PE reads observed for a *LMON*_*R5* (vs. a maximum of 591,000 observed for a single mRNA transcript). These results are in line with previous findings from other bacteria, from which asRNAs were detected for 20–75% of genes [35, 179–181].

Overall, more asRNA transcripts were upregulated than downregulated under cold stress (Fig 3). The largest number of upregulated asRNA transcripts was observed at G1 (Fig 3), which makes sense given that asRNAs act as gene regulators. At T1, cells are actively restructuring their transcriptional activity and cellular processes to prepare for growth in their new environment [175]. Around 10% of the asRNAs currently described for *L*. *monocytogenes* are thought to be involved in regulating transcription regulators [34]. This idea is supported by the overall low levels of antisense expression observed in this study.

While many asRNA transcripts were upregulated at G1, total antisense transcription was generally highest in late stationary-phase cells at both temperatures (Table 8), highlighting the importance of antisense regulation at this growth phase. Compared with regulatory proteins, asRNA transcripts offer the advantage of being able to provide a rapid connection between quorum sensing and direct destabilization of target mRNAs; moreover, as they can act post-transcriptionally, they can more tightly control the repression of proteins under environmental stress conditions [34, 182, 183]. Finally, unnecessary regulatory RNAs can be cleared quickly and via a process that requires less energy consumption than the removal of regulatory proteins. This may in part explain the abundance of asRNAs during late stationary-phase, in which energy sources are limited.

**Table 8:** Top 20 most highly expressed antisense transcripts in *L*. *monocytogenes* at 20°C and 4°C.

Overlapped EGD ORF	Description of EGD ORF	ORF strand	ORF length (bp)	Type of antisense transcript	% of ORF covered	Normalized paired-end read counts*									
						C1	C2	C3	C4	C5	T1	T2	T3	T4	T5
*LMON_0226*	Hypothetical protein	-	161	5’ UTRof *LMON_0227*	100	1693	1739	1200	730	618	738	1205	960	673	666
*LMON_0347*	Hypothetical protein	+	113	3’ UTR of *LMON_0348*	62	75	49	54	118	593	120	89	109	370	3838
*LMON_0384*^a^	Transcriptional regulator, DeoR family	-	920	3' UTR of *LMON_0383*	100	421	291	415	952	6745	816	449	494	1467	21581
*LMON_0652*^a^^b^^c^	Magnesium and cobalt transport protein CorA	+	950	5' UTR of *LMON_0651*	38	33	17	42	874	2380	289	242	344	1411	12635
*LMON_0681*^d^	Flagellar biosynthesis protein FliP	+	767	IT	100	12	18	107	1715	5087	69	169	256	603	1644
*LMON_0682*^d^	Flagellar biosynthesis protein FliQ	+	272	IT	81	2	8	69	1642	2650	40	121	218	442	422
*LMON_0738*^a^	Transcriptional regulator, XRE family	+	509	3' UTR of *LMON_0739*	100	205	336	321	406	289	1044	574	612	590	17856
*LMON_1143*	Propanediol utilization transcriptional activator	-	884	IT	100	239	120	818	1024	11556	222	106	176	241	364
*LMON_1780*^a^	Cell-shape determining protein MreBH	+	992	IT with *LMON_T39*	100	975	938	895	1403	2973	2575	1257	1311	1453	19114
*LMON_2216*	Hypothetical protein	-	665	Unclear	100	5	3	3	55	460	16	6	3	39	3567
*LMON_2351*	Hypothetical protein	-	443	3' UTR of *pepC*	100	115	108	183	307	1147	87	127	259	517	7613
*LMON_2352*	Pseudouridine 5'-phosphate glycosidase	-	911	3' UTR of *pepC*	100	86	61	66	68	624	107	107	156	293	7949
*LMON_2353*	Pseudouridine kinase	-	1118	3' UTR of *pepC*	100	61	48	43	37	442	102	81	98	179	6452
*LMON_2550*	Hypothetical protein	+	206	5' UTR of *atpI*	100	1932	3007	8743	17166	11120	631	2379	6384	15637	13462
*LMON_2699*^a^^b^	ImpB/MucB/SamB family protein	+	1256	3' UTR of *LMON_2700*	100	919	978	1240	1568	1422	873	841	968	1175	6461
*LMON_R1*	5S rRNA	+	1520	IT	100	1515	2188	4954	8066	9881	5305	5839	4863	6117	16314
*LMON_R2*	23S rRNA	+	2931	IT	100	3196	4648	10041	15825	19762	9955	11248	11037	14694	39471
*LMON_R4*	16S rRNA	+	1520	IT	100	1561	2162	5015	8089	9955	5323	5919	4922	6091	16331
*LMON_R5*	23S rRNA	+	2931	IT	100	3188	4578	10055	15765	19809	9819	11148	11018	14626	39964
*LMON_T39*	tRNA	+	71	IT	100	73	73	71	170	255	193	105	113	146	1458

* The normalized read counts presented represent the average of the 2–3 biological replicates. ORFs with the highest abundance of antisense transcription were determined by dividing the number of PE reads per ORF by the length of each ORF. ORFs highlighted in yellow have been previously shown or are assumed in the present study to be long antisense transcripts covering multiple ORFs. The following superscripts denote the study in which a transcript was identified:

^a^ Wehner et al. [38];

^b^ Wurtzel et al. [183];

^c^ Mraheil et al. [36];

^d^ Toledo-Arana et al. [31]. C1–C5 and T1–T5 represent the five growth phases at 20 and 4°C, respectively. Abbreviations: UTR = untranslated region; IT = individually transcribed.

Most genes with no or <10 asRNA transcripts encoded tRNAs (S2 Table). By contrast, rRNA had the highest levels of antisense transcription (Table 8). Similarly, Wehner et al. [38] have reported high levels of antisense transcription for rRNA in *L*. *monocytogenes* under intracellular growth conditions. Given the critical importance of ribosomes in cell functioning, it makes sense that bacteria would take advantage of the tight means of regulation offered by antisense transcripts. Other highly expressed antisense transcripts targeted genes encoding the cell-shape determining protein, MreBH; flagellar biosynthesis protein, FliP; magnesium and cobalt transport protein, CorA; and two transcription regulators (*LMON*_*0384*, *LMON_0738*), among others (Table 8). Again, most of these genes exhibited maximum levels of asRNA transcription in stationary-phase cells with higher levels evident at 4°C than 20°C.

**Fig 9:**
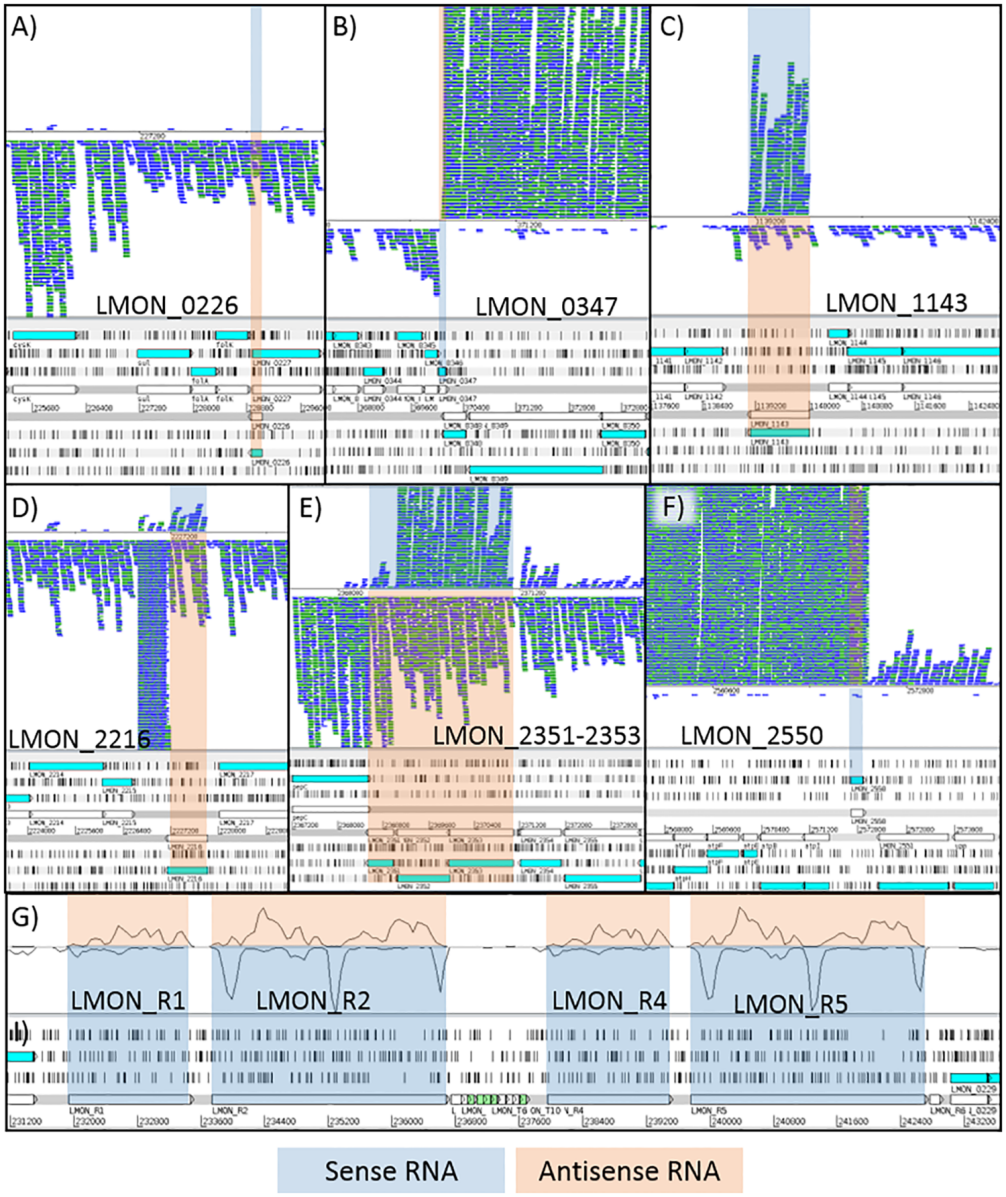
Coverage maps of select highly expressed antisense transcripts in *L*. *monocytogenes* cells grown at 20 or 4°C (refer to Table 6). The coverage maps shown are from stationary phase cultures grown at 4°C. Panels A-F shows individual paired-end, reads while panel G shows overall coverage trends for rRNA (5, 16, and 23S). Reads with the same start and end alignment positions are overlaid and depicted in green. Areas shaded in blue and orange denote sense and antisense transcripts of target genes, respectively.

Some *L*. *monocytogenes* genes exhibited high levels of antisense transcription with no or very low levels of mRNA transcription. Not surprisingly, this occurred when an antisense transcript was an extension of a 3’ or 5’ untranslated region (UTR) of an adjacent gene (Fig 9A, 9B and 9F), suggesting that the asRNA probably prevented RNA polymerase from binding to the promoter region of the gene on the opposite strand. On the contrary, antisense transcripts that appeared to be individually transcribed, frequently had high levels of mRNA expression (Fig 9E, 9G and 9M). These forms of asRNA have been shown to alter transcript stability by forming a sense/antisense RNA duplex leading to RNase-mediated degradation [183–186] or by stabilizing mRNA transcripts by inducing cleavage of unstable polycistronic transcripts [187–189]. Future research aimed at validating the mechanisms and functions of specific *L*. *monocytogenes* antisense transcripts will further enhance our understanding of gene regulation in this pathogen and possibly lead to the development of novel intervention strategies that can be used in the food industry.

## Conclusions

Here we present results from the first time-course study to investigate the transcriptional response and associated cold-induced membrane FA changes of *L*. *monocytogenes*, from early growth phases through late stationary-phase. To the best of our knowledge this is also the first study to look at asRNA expression in *L*. *monocytogenes* during cold stress and across multiple growth phases. Our results revealed that the *L*. *monocytogenes* transcriptomic response to cold stress is most active during late stationary-phase survival. Similarly, we show that the *L*. *monocytogenes* cold-stress regulon differs greatly across growth phases, highlighting the importance of carefully selecting appropriate time points when designing and conducting transcriptome studies.

Overall, more genes were suppressed than induced in *L*. *monocytogenes* under cold stress conditions. A core set of 22 genes was upregulated at all growth phases, including nine genes required for BCFA synthesis, the osmolyte transporter genes *opuCBCD*, and genes encoding the internalins A and D. This reflects the cell’s need to synthesize BCFAs to maintain membrane fluidity at low temperatures, and to support proper protein-folding through the uptake of structural-stabilizing osmolytes. Genes suppressed at 4°C were largely associated with cobalamin (B12) biosynthesis or the production/export of cell wall components. While is unclear how *L*. *monocytogenes* may benefit from downregulating cobalamin biosynthesis genes during cold stress, a reduced rate of peptidoglycan/cell envelope turnover at 4°C relative to 20°C may reflect the reduced cellular growth rate at this temperature, or allow carbohydrates to be conserved for alternative uses.

Notable cold-induced membrane FA changes included a 15% increase in the proportion of BCFAs and a 15% transient increase in UFAs between the lag and exponential phases. Such information may be useful for improving intervention strategies that target the food industry, as increased membrane UFA levels are known to increase the susceptibility of *L*. *monocytogenes* to salt and several antimicrobials.

Overall, around 70% of *L*. *monocytogenes* genes exhibited antisense transcription but only a small portion of antisense transcripts exhibited expression levels comparable to that of mRNA. On average, antisense transcription was higher at 4°C than at 20°C, highlighting the importance of antisense regulation in the *L*. *monocytogenes* CSR. The largest number of upregulated antisense transcripts was observed during early lag phase; however, at both temperatures antisense transcription was generally highest in late stationary-phase cells. Stationary-phase cells likely benefit from the tight control of protein expression that is offered by antisense RNA, thereby reducing the overall energy requirement of the cell while faced with nutrient-limiting conditions.

Collectively, our results reveal novel gene expression patterns and membrane FA alterations that occur in *L*. *monocytogenes* following cold stress, and highlight the abundance of antisense transcription in this microorganism at both 20°C and 4°C. We believe that this research will serve as a platform for future cold-stress studies by facilitating the selection of candidate genes and antisense transcripts for further functional validation.

## Supporting information

S1 Table Differential expression (log_2_) and associated cluster memberships of *L*. *monocytogenes* EGD ORFs and antisense RNA at 4°C vs 20°C. Genes belonging to a single cluster exhibited ≥0.50 membership. For ORFs with 0.2–0.5 membership to multiple clusters, the clusters are listed in decreasing order of membership. A * denotes ORFs with <0.5 membership to one cluster while a “0” denotes ORFs with <0.2 membership to one or more clusters. NC = average normalized count for control treatment.
(XLSX)

S2 Table Normalized paired-end read counts for all *L*. *monocytogenes* EGD ORFs and antisense RNAs at 4 and 20°C. Paired-end read counts were averaged across biological replicates. “as” denotes antisense RNA transcripts.
(XLSX)

S3 Table Fatty acid membrane profiles of L. monocytogenes at 4°C and 20°C. Values are presented as percentages relative to the total amount of extracted fatty acids.
(XLSX)

S1 Fig Correlation between differential expression (log2) levels obtained using RNA-sequencing and quantitative real-time PCR (qPCR). Points represent the average differential expression values for the genes *cspB* (▲) and *leuA* (●) at all five growth phases evaluated in this study. The y-axis represents the differential expression levels obtained using RNA sequencing while the x-axis represents the levels obtained using qPCR.
(TIF)
