# Supplementary figures and images for: Strand specific RNA-sequencing and membrane lipid profiling reveals growth phase-dependent cold stress response mechanisms in Listeria monocytogenes
Source: PLoS One. 2017 Jun 29;12(6):e0180123. doi: 10.1371/journal.pone.0180123 (PMC5491136; doi:10.1371/journal.pone.0180123)

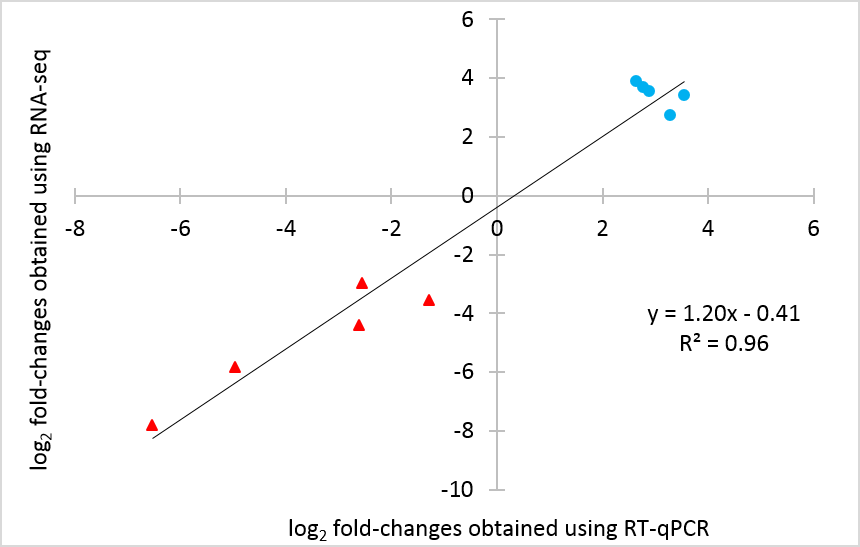

Supplement: S1 Fig — Points represent the average differential expression values for the genes cspB (▲) and leuA (●) at all five growth phases evaluated in this study. The y-axis represents the differential expression levels obtained using RNA sequencing while the x-axis represents the levels obtained using qPCR. (TIF) [file pone.0180123.s004.tif]
